# Evolutionary versatility of eukaryotic protein domains revealed by their bigram networks

**DOI:** 10.1186/1471-2148-11-242

**Published:** 2011-08-18

**Authors:** Xueying Xie, Jing Jin, Yongyi Mao

**Affiliations:** 1 Research Center for Learning Science, Southeast University, Sipai Lou 2, Nanjing 210096 China; 2 Centre for systems Biology, Samuel Lunenfeld Research Institute, Mount Sinai Hospital, 600 University Avenue, Toronto, Ontario M5G 1X5 Canada; 3 School of Information Technology and Engineering (SITE), University of Ottawa, Ottawa, Ontario, K1N 6N5 Canada

## Abstract

**Background:**

Protein domains are globular structures of independently folded polypeptides that exert catalytic or binding activities. Their sequences are recognized as evolutionary units that, through genome recombination, constitute protein repertoires of linkage patterns. Via mutations, domains acquire modified functions that contribute to the fitness of cells and organisms. Recent studies have addressed the evolutionary selection that may have shaped the functions of individual domains and the emergence of particular domain combinations, which led to new cellular functions in multi-cellular animals. This study focuses on modeling domain linkage globally and investigates evolutionary implications that may be revealed by novel computational analysis.

**Results:**

A survey of 77 completely sequenced eukaryotic genomes implies a potential hierarchical and modular organization of biological functions in most living organisms. Domains in a genome or multiple genomes are modeled as a network of hetero-duplex covalent linkages, termed bigrams. A novel computational technique is introduced to decompose such networks, whereby the notion of domain "networking versatility" is derived and measured. The most and least "versatile" domains (termed "core domains" and "peripheral domains" respectively) are examined both computationally via sequence conservation measures and experimentally using selected domains. Our study suggests that such a versatility measure extracted from the bigram networks correlates with the adaptivity of domains during evolution, where the network core domains are highly adaptive, significantly contrasting the network peripheral domains.

**Conclusions:**

Domain recombination has played a major part in the evolution of eukaryotes attributing to genome complexity. From a system point of view, as the results of selection and constant refinement, networks of domain linkage are structured in a hierarchical modular fashion. Domains with high degree of networking versatility appear to be evolutionary adaptive, potentially through functional innovations. Domain bigram networks are informative as a model of biological functions. The networking versatility indices extracted from such networks for individual domains reflect the strength of evolutionary selection that the domains have experienced.

## Background

Domains are the structural units of proteins that can independently fold and exert catalytic or binding activities. The majority of proteins are composed of one or more domains, with the exception of certain unstructured polypeptides. It has been widely recognized, for example, in the Structural Classification of Proteins (SCOP) database, that domains are also evolutionary units which have undergone duplication and recombination [1]. Domain shuffling/recombination, gene sequence duplication and divergence are three major mechanisms contributing to the evolution of organismal complexity [2]. Here organismal complexity means the number of cell types in an organism as defined by Basu et al. [3]. Thus, the network properties reflecting the interconnectedness of domains are important hints for understanding protein functions and proteome evolution. With the advent of numerous completely sequenced genomes, much research effort is focused on addressing the evolutionary mechanism that drives domain recombination and divergence [2,4-12]. In across-genome study, Apic et al. surveyed the domain combinations in 40 organisms ranging over three super-kingdoms and concluded that recombination of common domains has been the main contributing factor for the evolution of lineage-specific functions [5]. In addition, it has been shown that the phylogeny determined by protein domain profiles and domain combination profiles across genomes highly agrees with the taxonomic lineage relationships [7,9]. Several measures of domain's network properties were introduced to evaluate the ability of a domain to form different combinations [11,13]. Tools for analyzing complex networks have also been used in defining either the global protein domain networks [4,6,8], or other biological networks, such as metabolic networks [14], protein interaction networks [15], and gene regulatory networks [16]. Network analysis tools such as graph-theoretic analysis [6] and hierarchal clustering algorithms [12] were also adapted to compare domain organizations across multiple organisms. Wuchty et al. introduced the notion of "*k*-core" to the analysis of domain co-occurrence networks, where they compared sub-networks obtained via *k*-core decomposition with the corresponding physical protein domain interaction networks and argued that the driving force behind domain fusion is a collective effect caused by the variety, rather than the frequency, of the interactions [8].

On the other hand, focusing on understanding how new cellular systems arise, our recent large-scale cross-genomes study followed the evolutionary trajectories of domains [12]. In particular, we introduced the concept of "domain clubs", which are sets of proteins that share common domain compositions. The study revealed that evolutionary jumps are associated with a domain that coordinately acquires a new intrinsic function and enters new domain clubs, thereby providing the modified domain with access to a new cellular microenvironment [12].

These findings underlie the dynamic nature of domain evolutionary cycles between abrupt punctuation (domain shuffling) and equilibrium (domain modifications). As such, the network depicting the complex linkages among the domains in the modern-day genomes may be viewed as a consequence of such evolutionary cycling, in which both robustness against genetic perturbation and adaptivity to the micro-environmental changes are essential. At present, the biological mechanism for cells to acquire both robustness and adaptivity remains largely mysterious. Nevertheless one naturally expects that the mystery involves not only the biochemistry of each individual domain, if domains are considered as the evolutionary units, but also the domains' global organization and the inter-connectedness in the genome.

In the present study, we set out, on one hand, to establish a computational framework that may adequately reveal the biological mechanisms underlying cellular evolutionary cycles and, on the other hand, to probe such mechanisms. Via a survey of 77 eukaryotic genomes, we rationalize the use of domain bigram networks as a computational model for the study of evolution and our analysis indicates a hierarchical modularity phenomenon existing in domain bigram networks. Based on "*k*-core decomposition" analysis [8], we introduce the notion of "networking versatility" for individual domains to capture how each domain compromises in the network for both cellular robustness and adaptivity during evolution. We show that domains' networking versatilities correlate with their sequence conservation levels and with their biological functions. Domains having diverse versatilities and their hierarchically modular organization in a cell may serve as a simplified and yet insightful answer to the question how cells balance between their resilience to change and their flexibility to evolve.

## Results

### Domain Bigram as a Model of Linkage

A survey of 77 eukaryotic genomes (see additional file 1: Supplementary Results and Tables S1~S4 in additional file 2) in four kingdoms reveals that a relative small fraction of protein domains commonly exist in all species, presumably performing essential biological functions required in all living organisms. It is also observed that the majority of the kingdom-specific domain types are related within rather few biological processes, implying potential co-evolutionary ties therein. This work attempts to establish a new domain-linkage-based metric for the study of domain evolution.

A simple and first-order model of covalent linkage among domains is the linkage between a pair of distinct domains co-existing in a protein and closely situated as a duplex in the protein's amino-acid sequence. Such a domain pair is often referred to, in literature, as a "domain combination" [5,9,10,17] and sometimes as a "bigram" [11] -- a term borrowed from language modeling that refers to a tandem pair of words [18]. Here we prefer the term "bigram" to "domain combination" (due to the possible ambiguous interpretation of the latter) and call such a domain pair a *domain bigram*, or simply a *bigram*.

Domain pairs can be defined in other ways such as using domain co-occurrences [4,6,8]. We prefer domain bigram to domain co-occurrence in this study, rationalized as follows: (1) The notion of domain co-occurrence does not distinguish between domain architectures (N-terminal-to-C-terminal sequences of constituent domains in individual proteins) with same domain composition but different in domain ordering, which are usually generated by distinct evolutionary events [10]; (2) a single protein architecture consisting of a large number of domains can result in a large clique in the network graph, over-emphasizing the connectivity of domains therein, especially in multi-cellular organisms of eukaryotes [8].

Although comparing with higher-order linkage models, such as domain architectures, bigrams are rather simplified, they appear to serve as an adequate analytic tool for global studies of linkages within and across genomes (see additional file 1: Figure S2). For each genome, a "bigram network" can be constructed, where each vertex represents a domain in the genome and each edge connecting two domains represents a domain bigram that has appeared at least once in one protein. Under this framework, we constructed bigram networks for 77 eukaryotic genomes (refer to Table S1 in additional file 1), which we use to study the functional organization of genomes across species.

### Hierarchical Modularity of Bigram Networks

**Figure 1:**
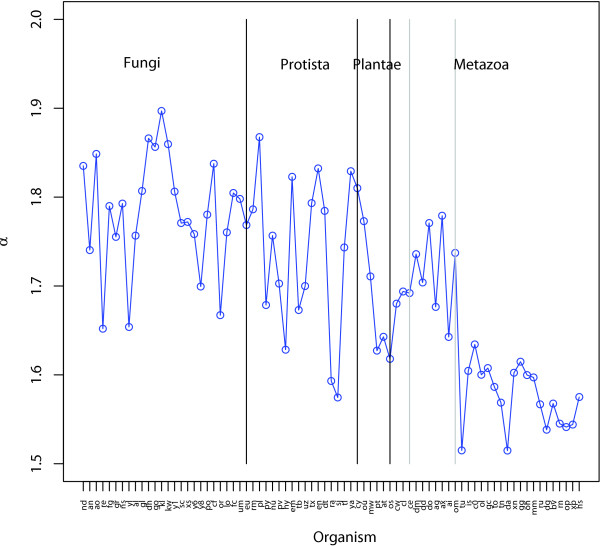
**Distribution of power-law parameter (α) of domain degrees across 77 bigram networks**. From the left to the right in the plot, organisms of the *x*-axis labels are ordered in protista, fungi, plantae and metazoa. In each studied organism, node degree in the bigram network follows a power-law distribution, *n*(*k*) ∝ k^*-*α^, where *n(k) *denotes the number of domains having degree *k*. The value *α *in the power-law distribution reflects to the overall likelihood that a bigram involving two randomly selected domains may exist. A comparison of *α *values across the 77 genomes suggests that overall trend that in more complex organisms such as in metazoa and plantae, a domain tends to have more chances to recombine with other domains.

For each of the 77 genomes, the distribution of the node degree in the bigram networks is observed to follow approximately the power law, the slopes of which in the log-log plots are consistent with organismal complexity (Figure 1). This observation is consistent with previous studies [10]. This indicates a "scale-free" nature of the networks - a common property of many large networks, which is typically inferred by a power-law distribution of the vertex connectivity [19-22].

We also computed the average clustering coefficient and average distance (see Methods) for each of the 77 bigram networks. The clustering coefficient of a network measures, on average, how well all neighbors of any vertex in the network are connected whereas the average distance measures how far the nodes in the network are separated apart. It has been studied that when the clustering coefficient is significantly higher than that of a randomly generated graph with the same size and edge density, the network exhibits a "modularity" [23]. Such modularity is also accompanied by small average distance.

**Figure 2:**
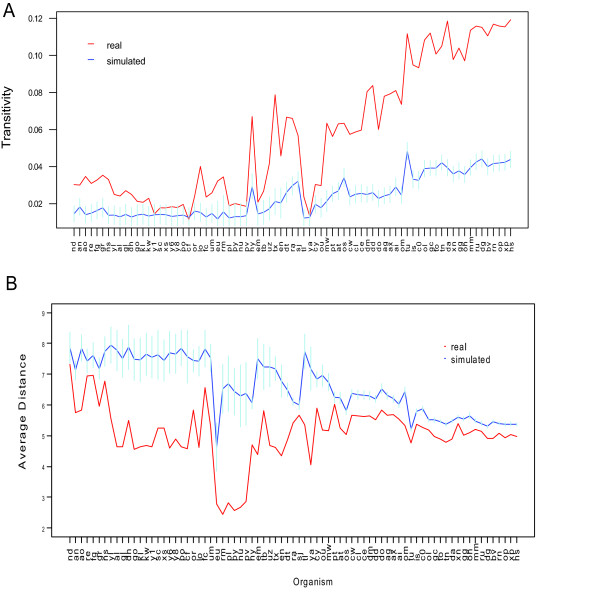
**Average clustering coefficient and Distance of the domain bigram network versus the simulated network**. A. Average clustering coefficient across domain bigram networks are plotted in red polyline along *x*-axis labeled with organism abbreviations (in the same order as in Figure 1). For the bigram network of each organism, 1000 simulated random networks with the same degree distribution are generated, and the mean and standard deviation of average clustering coefficient of simulated networks are shown in the figure with blue polyline (mean) and light blue vertical lines (standard deviation). B. Average distances of domain bigram networks and those of simulated networks, plotted in a similar manner as in A.

Overall, we observe that the average clustering coefficient of most species is significantly higher than those of the random networks (noting the value in general increases with species complexity (Figure 2A)) and that the average distance on the other hand is significantly lower than those of the random networks (Figure 2B). For higher species (such as metazoans), the differences in average clustering coefficient, relative to the corresponding random networks are particularly distinctive. This indicates that the bigram networks, and hence arguably the functional organizations of biological organisms, in general assume certain form of modularity. The increase of such modularity during evolution and its trend of diverging from random networks suggest that as species advance and diversify, their molecular functions assume increasingly refined and structured organizations.

The co-existence of scale-freeness, indicated by the degree power-law relationships, and modularity, indicated by high clustering coefficients deserves particular attention, since folk knowledge typically equates "scale-free" with "random", and "modular" with "structural". In fact, it has been observed that scale-freeness and modularity are not necessarily mutually exclusive; for example, they co-exist in some complex networks such as the metabolic network [20,23], protein interaction networks [14,19] and the world wide web [24]. We also observe a power-law dependence of clustering coefficients on node degrees in domain bigram networks (see additional file 3), *c*(*k*) ~ *k*^-γ^, with *γ *= 1.63 ± 0.46. As established by Ravasz and Barabasi [25], the dependence of clustering coefficient on the node degree is an indicator of what is known as "hierarchical modularity": the network nodes are randomly connected to form modules and modules are randomly connected for form larger modules; this process repeats to form the entire network.

**Figure 3:**
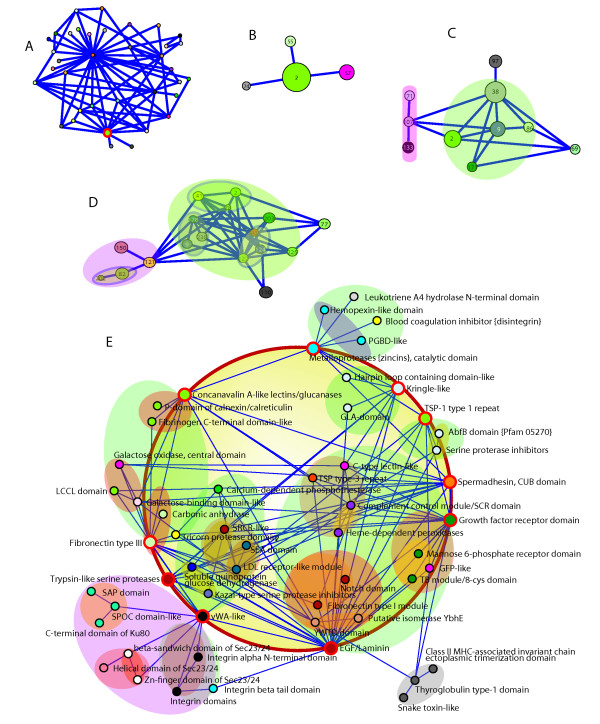
**Hierarchical organization of domain bigram network in *H.sapiens: *a case study**. Using a topological overlap matrix [23], domain bigram network in *H.sapiens *is divided into 123 modules after average-linkage hierarchical clustering and thresholding the clustergram at 0.95. A. the largest connectivity component of domain bigram network is plotted, where each module is suppressed to a node. The node circled with red color is selected as an example to illustrate the hierarchical organization of the modules. B. The node selected in A can be divided into four modules (2, 55, 57, 74; node size indicating the abundance of domain types within the node, or module) when the clustergram threshold is set to 0.90. C. When the clustergram threshold is set to 0.7, a module is further subdivided into several smaller submodules. D. The organization of the modules when setting threshold to 0.5. E. When the threshold is set to 0.3, most modules split into groups that consist of no more than three domains (SCOP annotations). The "parent" module of a sub-module is represented by a color-coded underlying "plate" and the color code of a module is consistent across all panels.

From this perspective, our results suggest that modules in domain bigram network are organized by such a hierarchical principle. As argued in Ravasz et al. [23], such a hierarchical modularity (see Figure S4 in additional file 1 and Figure 3 for detail illustration) may have important implications of evolution: local changes of bigrams, as consequences of gene recombination, can be absorbed and yet accumulated during evolution, which on one hand makes the species robust to deleterious impact and on the other hand serves as a genetic basis for the species to progressively advance. See panel E in Figure 3, a red circle is added to connect the high-degree nodes. The domains on the circle may each be viewed to serve as a "gateway" of the larger module it resides in. Here, a network "gateway" loosely refers to an internetworking system capable of joining together two networks that use different base protocols. As this sub-network in panel E appears to comprise mostly extracellular domains, functional modularity of these domains within the entire proteome is apparent. Take the blue module at the top of panel E for example. The gateway domain for this module is the Metalloproteases domain, which catalyses the degradation of the extracellular matrix. Within this module, there is the structurally similar (and possibly related) PGBD-like domain, which has a general peptidoglycan binding function, together with the Blood coagulation inhibition domain, Leukotricene A4 hydrolase domain and Hemopexin-like domain. The latter three domains in their constituent proteins are involved in crucial aspects of hematopoiesis. This observation suggests that the network is to some extent structured according to functional relevance at various levels of the hierarchy. This is consistent with the understanding that these domains in their constituent proteins had co-evolved, and thus supports the notion that the complexity of biological networks has been increased in a modular fashion. Additional example at the lower-left (on a purple background) is a module through a vWA-like gateway domain (vWA for von Willebrand/integrin A), which represents a family of widely dispersed domains with roles in cell adhesion and elsewhere. It is connected to two smaller modules, the orange one on the top and the blue one at the bottom. Within the blue module are subfamilies of Sec23/24 domains, all sharing a similar structure with (and possibly related to) vWA domain. To their right is the family of integrin cell matrix domains. These domains are linked in their constituent proteins, which form the matrix infrastructure in multicellular animals. These example modules reveal that some domains exert certain "gateway-like" functions in the network, potentially having led to a stepwise expansion of a new module of specialized lineage functions.

### Domain Networking Versatility via k-Core Decomposition

The existence of hierarchical modularity of bigram networks necessarily implies that some domains in the network appear more "versatile" than others and serve to connect the otherwise distant modules with rather distinct functions. We note that the term "versatility" here not only describes a domain's tendency to join other domains but also captures the domain's contribution to the global connectivity of the network. For this purpose, we introduce a notion of versatility, which we call "networking versatility", based on a computational technique known as "*k*-core decomposition" [26]. More specifically, we define the networking versatility index (or versatility index) of a domain as the largest value of *k *such that the domain is contained in the *k*-core of the bigram network. In a sense, domains with high versatility index are situated in the "core" of the network, whereas those with low versatility index are situated in the network "periphery". This shares certain similarity with the notion of local and global centrality of interacting proteins in protein interaction network [27].

**Figure 4:**
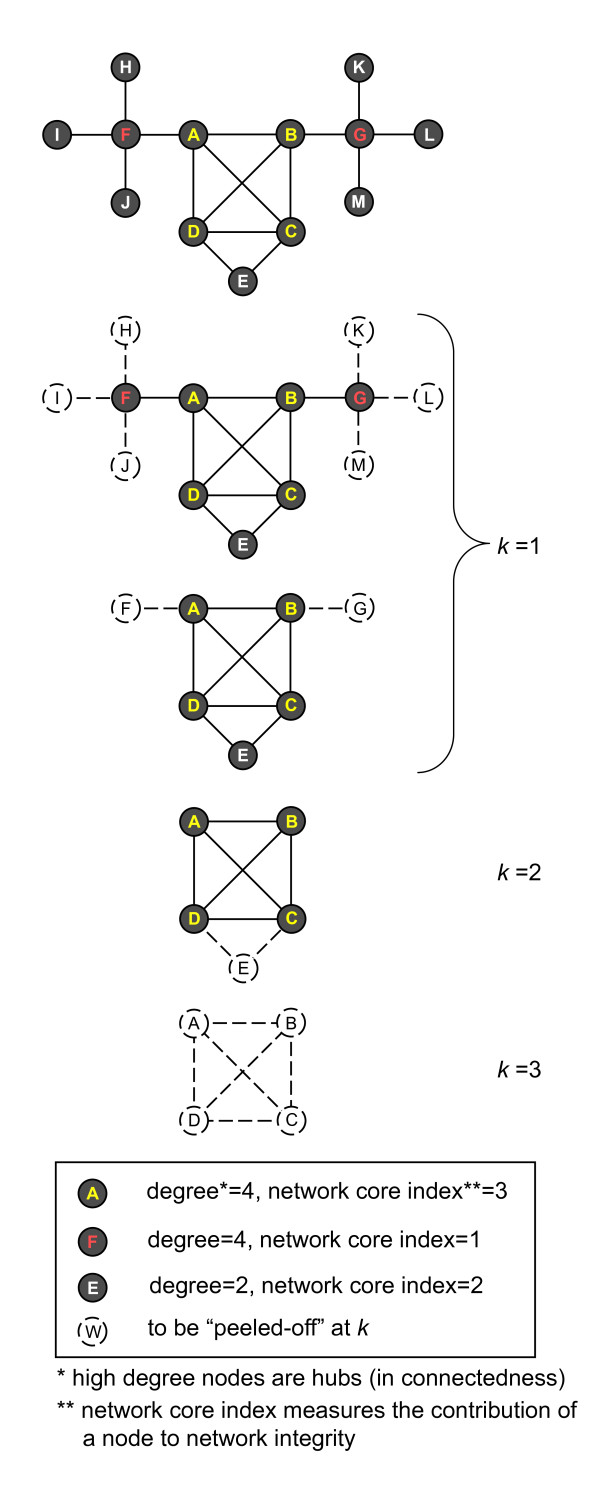
**The greedy procedure of *k*-core decomposition**. The *k*-core decomposition procedure is illustrated with the toy example (from top to bottom, *k *= 1, 2, 3). Note that nodes with high degrees do not always reside in a *k*-shell with large *k *(see the comparison of nodes A, F, E and I for that matter in the legend box). Taking nodes F and G for example (in red), despite the fact they have the highest degree of connectivity (4) and are directly connected to the core nodes (A-D in yellow), they have the lowest *k *value (1). This indicates that via *k*-core decomposition, F and G are rated as peripheral hub nodes that have low contribution to the global integrity of the network.

We note that other notions of domain versatility or promiscuity have been studied previously [11,13,28], for example, in terms of the degree of a domain in a properly constructed network. Such notions of versatility are usually limited to the local connectedness of the domain and are less directly related to the global network connectivity. The difference between versatility index introduced here and other versatility notions defined in terms of vertex degree is demonstrated in Figure 4 via the difference between vertex degrees and versatility indices. In fact, as shown in Figure 4, nodes with high degrees or even directly connected to the core nodes, if having a low versatility index, constitute peripheral hubs. In addition, the term versatility here is intended to emphasize the overall contribution of a domain to the global network integrity and, as will be shown later, largely indicates the evolutionary pressure that the domain has experienced. The broadly adopted notion of domain promiscuity on the other hand reflects more the intrinsic propensity (or chance, in coping with selection pressure) of a domain in forming multidomain proteins with other domain types. This fact suggests that a domain type with a large number of copies in the genome, although having more chance to participate in bigrams, is not necessarily a "core" domain. We caution that the k-core decomposition analysis does not take the domain abundance into consideration due to its inherent limitation, namely, that it is suited only for unweighted networks. Therefore, to measure the "full" contribution of a domain to the overall network integrity, an enhanced technique beyond k-core decomposition is perhaps needed.

We construct a bigram network for each of the four kingdoms, where the proteins considered for each kingdom is the union of all proteins in all studied species belonging to that kingdom. We apply *k*-core decomposition to each kingdom bigram network and obtain the networking versatility index for each domain therein. There are 10 nested cores in metazoa network (see Figure S3 in additional file 1 for visualization), 6 in plantae network, 5 in fungi network and 8 in protista network. In all four cases, we noticed that as *k *increases, the largest connectivity component in the *k*-core remains connected. This suggests that rich redundancy has been built in the network, particularly in the "main" component, in the sense that there are more than one ways the Nature may choose to use a domain in a different functional module.

We identify the domains residing in the innermost core, i.e., those having the highest networking versatility index, in at least one of the four kingdoms and compare their versatility across the four kingdoms. Table S2 in additional file 1 lists these domains (in total 54 domains), among which 39 domains are in the innermost core of metazoa network, 17 in plantae network, 18 in fungi network and 15 in protista network. We observe that most domains contained in the innermost cores of lower kingdoms remain in the innermost core of the metazoa network. In addition, the domains in the innermost core of metazoa include certain metazoa-specific domains as well as some domains shared in other lineages (for example, "Immunoglobulin" and "Kringle-like" domains). These domains are markers of animal lineage and responsible for animal-specific blood coagulation system, immune response and apoptosis regulation to maintain cell viability. There are six domains shared by the innermost cores of all four kingdoms and 12 domains shared by the innermost cores of at least two kingdoms. We refer to these 18 domains (see domains with Interpro IDs labeled with * in Table S2 in additional file 1), the most versatile in certain kingdoms and across kingdoms, as the "core domains" for convenience. A closer look at the functions of the core domains reveals that these domains possess rather "universal" functions and play biochemical roles in a broad range of biological processes. Examples in this category include PH (Pleckstrin homology) domains and RING Finger domains. The functional universality of these core domains suggest that they, from a network perspective, play an important role in "connecting" the modules at various levels of the hierarchy.

It is also interesting to examine the domains that are the least versatile. These domains, not involved in any bigram and hence not contained in any core of the bigram networks, have versatility value equal to 0. The collection of such domains includes the domains that exist only as single-domain proteins and domains found in multidomain proteins but with at least 30 amino acids apart from other domains. These domains, which we call "network peripheral domains", each appear to be only involved in a narrow and yet essential family of cellular functions, known examples including Actin in structuring cytoskeleton and Histone in chromatic organization (discussed later). The contrast of functions between the core domains and the network periphery domains justifies the term "networking versatility" in the sense that this notion of versatility indeed captures a domain's ability to act across different functional subsystems.

### Correlation between Networking Versatility and Evolutionary Adaptivity

The distinct networking versatilities of domains observed above are arguably consequences of evolutionary selection. As such, a domain's versatility is expected to correlate, to some extent, to its evolutionary adaptivity, which may be reflected via its level of conservation sequence-wise or structure-wise.

**Figure 5:**
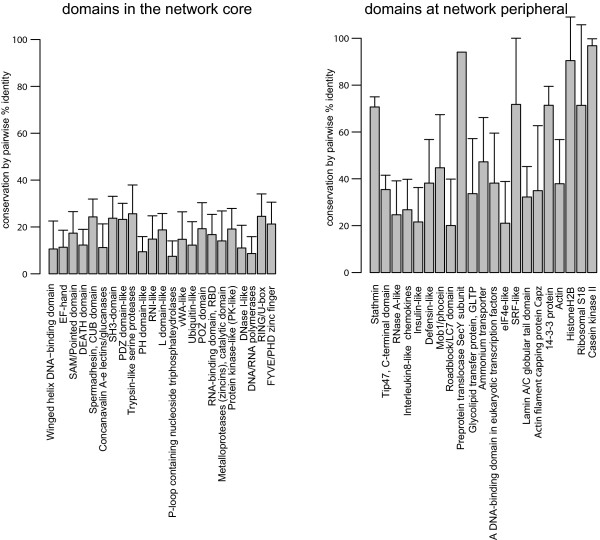
**Domains in the network core are less conserved than peripheral domains**. Human domains from the network's inner-most core (all at k = 10, left panel), excluding those comprised entirely of short repeating units in tandem and of varying spans, are measured for the level of sequence conservation within each domain family. The degree of conservation among all individual members of a domain family is presented as the mean and the standard deviation (error bar) of pair-wised amino acid identity (y-axis, in %) after alignment.

We use amino-acid identity to measure the degree of sequence conservation within a domain family. Specifically, multiple-sequence alignment of all members within each human domain superfamily was conducted and the amino-acid identity percentage for every pairing of two domains was obtained. We plotted the mean and standard deviation of the identity percentage for all core domains and for all network peripheral domains (Figure 5). The result suggests that the sequences of the core domains overall exhibit low degrees of conservation whereas those of network peripheral domains are much highly conserved.

### Assessment and comparison of functional evolutions of network peripheral domains and network core domains

As is shown above, conservation at the overall amino-acid sequence level may be treated as an "adaptivity score" for individual domain families, accumulated through their evolutionary history. Further assessing and measuring such adaptivity at functional levels are arguably more relevant. This is because it is conceivably the underlying functions of domains that have been more directly selected by the evolutionary pressure and via their constituent proteins. From a functional perspective and with respect to the network core domains and network peripheral domains, a question naturally arises as to whether these two classes of domains exhibit distinct adaptivity level.

**Figure 6:**
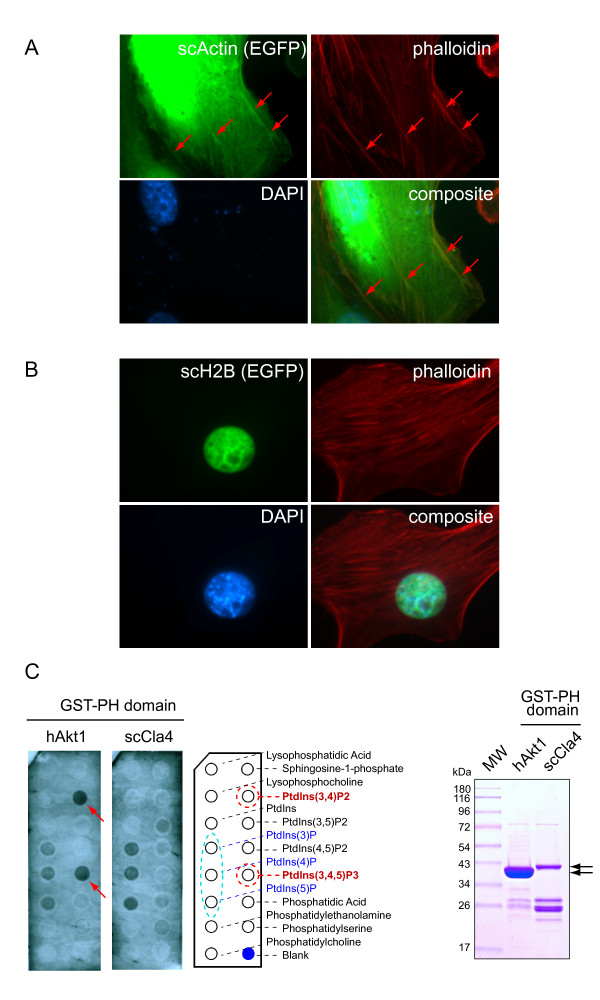
**Functional comparison between yeast and human domain examples**. Domain examples of Actin (A) and the histone core (B, scH2B) are chosen from the network peripheral domain category and PH domains (C) from the network core domain category. A. Yeast Actin (scActin) as an EGFP fusion protein was artificially expressed in the mouse NIH-3T3 fibroblast cells. Cells were stained with phalloidin (Texas-Red) and DAPI for polymeric F-Actin stress fibers and DNA (marks nucleus) respectively. Arrows in EGFP panel mark the "cable-like" distribution of scActin in the structures of polymeric Actin stress fibers illuminated by phalloidin. B. Similarly, the yeast histone protein (scH2B), also as an EGFP fusion, was expressed in NIH-3T3 cells. The expression of scH2B is completely restricted to the cell nucleus (marked by DAPI, and in composite panel). C. PH domains of human Akt1 (hsAkt1) and yeast Cla4 (scCla4) were purified as recombinant GST-fusion proteins. These proteins were used to probe the membrane-type PIP-strips in a protein-lipid overlay assay. Binding of PH domains to specific types of PtdIns-phosphates (left panels -- refer to the diagram and list for spot positions) was visualized through anti-GST-HRP far-western blots. Red arrows point to the binding of PH domains of hsAkt1 to PtdIns(3,4)P2 and ptdIns(3,4,5)P3 (highlighted in red circles in the diagram). Circled in light blue are the PtdIns-mono-phosphates that have no signaling roles in cells. Right panel: visualization of recombinant GST-PH domains of hsAkt1 and scCla4 (pointed by black arrows) on a coomassie blue gel.

We designed experiments on selected domain families to investigate the level of their functional conservation within the family. We focused on PH domains (Pleckstrin-homology domain superfamily) from the core, and Actin (Actin-like ATPase superfamily) and Histone (Nucleosome core histone superfamily) from the network peripheral domain category. For each of these domain types, we developed assays to compare the functions of two domains within the same type but from distant species, yeast and human in this case (Figure 6).

For Actin and Histone, given both domain types exhibit high degrees of similarity between their yeast and human sequences (see Figure S5 in additional file 1), we anticipated the yeast variants, once artificially expressed in mammalian cells, to occupy the subcellular compartments designated for their mammalian counterparts, i.e. cytoskeletal and chromatic structures respectively. Such observation would indicate that the yeast domain may serve as a functional replacement of the mammalian domain, and that the domain family has been under the evolutionary pressure against divergent adaptation. This hypothesis was experimentally tested as follows. We cloned alpha-Actin and Histone (H2B2) of *S cerevisiae *as EGFP (enhanced green fluorescent protein) fusions in a mammalian expression vector. These constructs were individually transfected into NIH-3T3 mouse fibroblast cells. The expressions of the yeast Actin (scActin) and Histone (scH2B) were visualized in the confocal EGFP channel (Figure 6A-B, upper-left). Meanwhile, phalloidin (conjugated with texas-red) and DAPI were used as counter-stains to mark the polymeric F-Actin stress fibers (upper-right panels in A and B) and nucleus (lower-left panels) respectively. Co-localization of scActin with the cytoskeletal structures marked by phalloindin was evident (in A). It was also observed that the human Actin (as mCherry conjugate) and EGFP-scActin co-localize (results not shown) in the cable-like structures in the cells (reminiscent of F-Actin stress fibers). With respect to the yeast scH2B expressed in mammalian cells, an exclusively nuclear localization of the protein is observed (in Figure 6B), consistent with its essential structural function of structuring nucleosomes in yeast. Furthermore, confocal images also revealed that not only the yeast and human H2B proteins were co-localized perfectly together in NIH-3T3 cells, their nuclear distributions interestingly contrast DAPI staining (see additional file 1: Figure S6), which is more selective for transcription inactive regions of the heterochromatins [29]. This observation indicates that both human and yeast H2B proteins were assembled into euchromatic nucleosomes at a faster rate (than into the heterochromatic nucleosomes). This manifested, with finer details, the remarkable level of functional conservation between these yeast and human H2B proteins.

As for the PH domains, since they are found in proteins with a broad range of domain architectures, some members tend to follow a rather versatile localization pattern depending on the cell's state of activation. It is therefore often difficult to interpret the functional significance of their subcellular distributions. The best characterized examples of the family are those found in serine/threonine kinase Akt that are known to bind to PtdIns(3,4)P2 and PtdIns(3,4,5)P3 (PIP2/3) in mediating Phosphoinositide 3-kinase signaling [30]. We therefore compared the phospholipid binding profiles of the PH domain from serine/threonine kinase Cla4 of yeast to that of human Akt1. At this point it should be noted that the yeast lacks the Phosphoinositide 3-kinases, hence implying a lack of evolutionary pressures for its PH domains to adapt their binding affinities to PIP2/3. The idea of comparing the PH domains of Cla4 of yeast to those of human Akt1 proteins was based on the fact that they, respectively, represent the only family of serine/threonine kinases that harbors PH domains in the two respective species (see domain architectures in additional file 1: Figure S8). This careful choice of PH domain pairs then justifies this experimental comparison, irrespective of the broad range of sequence divergence within PH domain family known to exist across many species.

Finally, we employed a standard assay to compare phospholipid binding properties of this pair of PH domains, purified both PH domains as GST-recombinant proteins (shown on gel in Figure 6C, right panel), and applied them in parallel to a pair of PIP-strip membranes spotted with various lipid types. Remarkably, while Akt1 PH domain displayed strong binding affinity to PtdIns(3,4)P2 and PtdIns(3,4,5)P3 (together referred to as PIP2/3), there was no binding to these lipids by the PH domain of Cla4 (Figure 6C, left panels, in red). As an important control for folding and function, both PH domains demonstrated "basal affinities", as expected, to all three forms of the phosphatidyl inositol monophosphates (Figure 6C, circled in blue). This experimental observation supports the notion that the PH domains have undergone divergent evolutionary paths, with an ancestor PH domain of Akt1 adapted and having since maintained its functions to recognize PtdIns(3,4)P2 and PtdIns(3,4,5)P3. This is possibly attributed to the emergence of Phosphoinositide 3-kinases in higher eukaryotic species. We note that the overall sequences of PH domains of Cla4 and Akt1 are very different (see additional file 1: Figure S7, upper panel). By using the tertiary structure of human Akt1 PH domain determined by crystallography [31] as a template, we were able to model its difference from that of the yeast Cla4 protein. Notably, many critical residues forming the phospho-lipid binding pocket for Akt1 are absent from Cla4 (see additional file 1: Figure S7, comparing the blue pocket in Akt1, left, with a modeled conformation with Cla4 amino acid replacements, in purple - right panel), indicating that these two PH domains have adapted and acquired distinct biophysical properties in phospho-lipid binding. This is consistent with the finding that the overall sequence homology between PH domains of yeast (in Cla4 and Skm1) and those of human (in Akt1-3) is low, contrasting the high degrees of sequence similarity between human and yeast domains in the cases of Actin and Histone (see additional file 1: Figure S5).

At this end, we have shown, not only from sequence conservation perspective but also from functional perspective, that network core domains and network peripheral domains exhibit distinct evolutionary adaptivities. Although our studies have only involved the two extremes of the "versatility spectrum" and finer analyses are desirable, these results have provided evidence indicating a correlation between evolutionary adaptivity of domains with the networking versatility introduced in this paper. Here we stress the importance of this concept of versatility, which we believe to set a stage for a comprehensive overview of domain evolution landscape. These results, at the same time, further justify the usefulness of domain bigram networks.

## Discussion

### Hierachical principles of domain bigram networks

In this work, we established domain bigram networks as a computational model for the study of cellular evolution. Via a survey of 77 eukaryotic genomes, we observed that domain bigram networks share common topological features, particularly scale-freeness, high average clustering coefficient and power-law relationship of clustering coefficients on node degrees, suggesting a hierarchical and modularized organization of the networks. Networking versatility index, defined based on k-core decomposition, is introduced to quantify the adaptivity of individual domains during evolution. It is observed that the networking versatilities of the domains correlates with their sequence conservation levels and with their evolutionary adaptivities measured experimentally. The hierarchical modular organizations of the domains in the cells and the ladder of the networking versatilities partially explain why a cell can be simultaneously robust against genetic perturbations and adaptive to micro-environmental changes.

Our survey of 77 eukaryotic genomes shows that profiling of species based on their bigram networks strongly correlates with the taxonomy classification, and this suggests that such networks may adequately serve as computational model for genomic linkages. The 77 bigram networks share common topological features, particularly scale-freeness, high average clustering coefficient and low average distance, suggesting a hierarchical and modularized organization of the networks.

### The modular platform for phosphotyrosine signaling led by the "gateway" function of SH2 domains

Based on these findings, it might still be premature to articulate the evolutionary forces in promoting the networks to adopt hierarchical and modular principles. Therefore, instead of attempting to conceptualize a general theory for domain organizations, we here use the example of the evolution of multicellularity promoted by phospho-tyrosine (pTyr) to elaborate on our point. An effective pTyr mechanism is comprised of a three-part system that requires the cooperation among the writers (tyrosine kinases - TyrK - that catalyze phospho-transfer reactions through ATP hydrolysis), the readers (such as Src Homology 2/SH2 domains that bind to phospho-tyrosines) and the erasers (tyrosine phosphatases -- PTP) (We note here that the SCOP database at its superfamily level doesn't distinguish TyrK from other kinases, nor PTP from other phosphatases -- thus the topic on this three-part domain system was not address in Results.). Such multi-component signaling platform is the hallmark of multi-cellular animals, and is suggested to result from stepwise evolution during the emergence of metazoan species [12,32]. From this perspective, one may consider these domains and their constituent proteins constituting such tripartite system a functional module, or "toolkit". It is interesting to note that at the domain level, SH2 domains, as readers of pTyr, are also frequently linked to TyrK and PTP domains in higher animals [12]. From a domain bigram point of view, these SH2 domains represent the "gateway" (discussed in Results and Figure 3) to the modular pTyr signaling platform. This allows us to speculate about the evolutionary role of the gateway SH2 domains. Evidence suggests that the tyrosine-binding SH2 domains appeared earlier than TyrK in the history of life. It is plausible that the first TyrK arose from a serine/threonine kinase (STK) or a dual-specificity kinase ancestor. Thus, it is striking to observe that the modern day slime mold *Dictyostelium discoideum*, which lacks TyrK, has the bigram consisting of SH2 and serine/threonine kinases (STK, or dual-specific STK, in Shk proteins), which resembles the SH2-TyrK bigram in the animals. This makes one wonder if the equivalence of such bigrams had occurred in species of the pre-TyrK world, and if the first *bona fide *TyrK would have arisen from an ancestor STK? This hypothetical scenario of evolutionary history illustrates how a gateway domain (such as SH2) potentially leads to modular expansions that contribute to the transition and advancement of lineages (See legend of Figure 3 for details on other observed "gateway" domains). As for the kinase domains, such STK-to-TyrK transition of catalytic specificity, which would certainly rewire the signaling circuitry of cells, further exemplifies the versatility and adaptivity of the domain networks.

### Implications of hierarchical modularity on evolution

Hierarchical modularity, often observed in social networks, plays an important role in the network's robustness to embrace and absorb impacts, namely those chromosomal changes resulting in aberrant domain linkages or amino acid permutations, which alter biochemical properties of domains and proteins. We infer that such hierarchical modularity in domain bigram networks and their encoding genomes also provides the cells with flexibility against punctuated impacts. In other words, the network has been compounded under the selection pressure in a fashion that is suited to buffer perturbations during domain recombination.

The scheme of k-core decomposition of bigram networks reveals that hierarchical modularity is associated with varying levels of networking versatilities of the domains in the networks, and that domains, during their evolutionary history, have diverse levels of adaptivity. The highly adaptive domains become capable of participating in distinct molecular contexts, acquiring new functions, and recombining with domains that are otherwise irrelevant. On the other hand, the non-adaptive domains conserve their specific functions during evolution. This perspective is demonstrated by the sequence conservation levels of core domains (the most versatile) and network peripheral domains (the least versatile). In particular, it is observed that the network peripheral domains are significantly more conserved than the core domains. In fact, the notion that some domains are adaptive (core) and some are robust (periphery) is analogous to the distinction between local and global notions of centrality of interacting proteins as pointed out in Wuchty and Almaas [8,27]. Our additional experimental analysis of PH domains (core domains), and Actin and Histone (network peripheral domains) further demonstrates the correlation between networking versatility and evolutionary adaptivity, where the two network peripheral domains retain their conserved functions (that have spanned across the evolution, namely, between yeast and human) to maintain certain basic and essential cellular operations. This contrasts the PH domains from the core of the networks. These domains, upon duplication, may tend to follow relaxed evolution on their paths towards divergence. During the process, new functions arose in phospho-lipid-binding, and that had possibly promoted the emergence of complex regulatory mechanisms, such as cell signaling through the PIP2/3 second messengers.

## Conclusions

The coherence of these results on one hand justifies the usefulness of domain bigram networks as a computational framework for the study of cellular evolution, and on the other hand reveals, to a degree, the biological basis underlying evolutionary cycles. We observed that the domain bigram network exhibits hierarchical modularity, which may serve as the basis for various functional platforms. Careful overview of the hierarchy and in-depth examination of the modules in domain bigram networks shall shed lights on how cells compartmentalize their functions at various levels of granularity and how these functions collaborate and interact locally and globally. Networking versatility, which is indexed by k-core decomposition of the network, appears to be a notion properly reflecting the evolutionary adaptivity of domains, as measured by sequence and functional conservation.

## Methods

### Superfamily domains assigned to eukaryotic genomes

This study used domain definitions from the SCOP database. SCOP first organizes domains into families if domains have a common origin supported by observable sequence resemblance. Families without observed sequence-level similarity are then clustered together into superfamilies if there is structural or functional evidence strongly supporting that they originated from a common ancestor. The SCOP hierarchy considers folds and groups superfamilies not known to be related but having the same secondary structures. The notion of SCOP superfamily, instead of family or fold, is taken as the definition of "domain" in order not to risk being too broad or too narrow [5,7]. We note that the classification of superfamily largely results from highly automated algorithmic procedures, where inaccuracy and omissions are frequent. Thus, expert assignment and verification remain crucial and often preferred in the study of specific domain families [33].

Seventy-seven eukaryotic organisms with completely sequenced genomes in the SUPERFAMILY [34] database are included in our analysis. Table S1 in additional file 1 summarizes the taxonomic information of these organisms together with the number of proteins in each organism and the fraction of proteins with domain assigned. It is worth noting that, unlike other three kingdoms, protista is a paraphyletic group and consists of organisms which cannot be classified into any fungi, plantae or metazoa. Domain bigrams in each genome are also extracted from the SUPERFAMILY database, in which proteins are annotated by superfamily domains with an expert-curated set of profile hidden Markov models. A domain bigram, consistent with the definition of domain combination in SUPERFAMILY, is defined as an adjacent domain pair with gap sequence less than 30 amino acids. This will avoid harboring any unknown domain [5]. Domains never having other partner co-occurring within 30 amino-acid distance are referred to as network peripheral domains in the paper.

### Network analysis

The versatility of a domain in its local context can be parameterized by the degree of the vertex in the network, which is defined as the number of vertices to which it links [28]. The clustering coefficient [35] of a vertex *i*, is defined as:

$$C\left(i\right)=\frac{2n_{i}}{k_{i}\left(k_{i}-1\right)}$$

where *n_i _*counts the number of edges between neighboring vertices of vertex *i, k_i _*is its degree. The average clustering coefficient of a network is defined as the mean value of vertex clustering coefficient.

The average distance (i.e. the mean shortest distance) in a network is defined as $l=\frac{2}{n\left(n+1\right)}\underset{i\geq j}{\sum }d_{ij}$,

where *d_ij _*is the shortest distance from vertex *i *to vertex *j *[35].

To explore the hierarchical topology of domain bigram network, we first computed the topological overlap matrix of the network according to the method in Ravasz et al. [23]. We then generated the distance matrix based on the topological overlap matrix and applied average-linkage clustering to obtain a dendrogram of domains. Applying cutoffs to the dendrogram, respectively at 0.95, 0.9, 0.7, 0.5 and 0.3, we gradually "peeled" the nested modules at different levels (see Figure S4 in additional file 1 and Figure 3).

Pajek, a program for large-network analysis, was used for the calculation and visualization of domain bigram networks. This program is available at http://vlado.fmf.uni-lj.si/pub/networks/pajek/.

### K-core decomposition of network

Following [26], for a given non-negative integer *k *and a given network, we define the *k*-core of the network as the maximum induced subgraph with vertices having degree not lower than *k *[26]. A greedy algorithm may be used to identify the *k*-core of a network by recursively removing all vertices with degree less than *k *until no such vertex exists in the resulting graph. It is possible that vertices with degree larger than *k *in the original network not be present in *k*-core since their degree could be smaller than *k *after their neighboring vertices are deleted (see Figure 4). Methodologically, *k*-core decomposition (namely, the procedure of obtaining the *k*-core for all *k*'s) serves as a non-parametric method to investigate whole-genome or multi-genome bigram networks.

Multiple sequence alignment and subsequent calculation of pair-wise amino acid conservation within individual domain superfamilies were executed using tools provided by ClustalW2 (http://simgene.com/ClustalW).

### Experimental methods

#### DNA constructs and reagents

Yeast Actin and Histone sequences of their respective encoding protein sequences were amplified from genomic locus of ACTIN1 and HTB1 respectively using the following PCR primer pairs:

5'-ATATGGCGCGCCATGGATTCTGAGGTTGCTGCTTTGG and 5'-GCTGATCGTTAATTAATTAGAAACACTTGTGGTGAACGATAG for ACTIN1, and 5'-ATATGGCGCGCCATGTCTGCTAAAGCCGAAAAGAAACC and 5'-GCTGATCGTTAATTAATTATGCTTGAGTAGAGGAAGAGTAC for HTB1/scH2B.

EGFP fusions were obtained from the Creator system (BD Biosciences) following manufactory instructions. PH domains were cloned from yeast Cla4 (amino acids 42-197) and human Akt1 (amino acids 1-127) using primer pairs: 5'-ATATGGCGCGCCACCAAACTTATGAGTCAACTGGATTTA and 5'-GCTGATCGTTAATTAATCAACCAACGTGAACTTTATGTGTGAAG, ATATGGCGCGCCATGAGCGACGTGGCTATTGTGAAGG and 5'-GCTGATCGTTAATTAATCAGTTGTCACTGGGTGAGCCCGACCG, respectively.

Phalloidin-Texas-red and DAPI were from Invitrogen. NIH-3T3 cells were from ATCC. Recombinant PH domains were harvested from pGEX 4T-2 expressions following the standard GST purification protocol (BD Biosciences). PIP-Strip™ was from Echelon Biosciences Inc. Polyclonal Anti-GST antibody was from GE Healthcare Life Sciences.

#### Transfections and cell imaging

NIH-3T3 cells were maintained in DMEM with 10% FBS. The transfection experiments were mediated by Lipofectamine 2000 reagent from Invitrogen. Twenty-four hours after the transfection, the cells were fixed in 4% PFA, permeabilized in 0.1% triton, and subsequently stained with phalloidin and DAPI. The confocal images were taken using an Olympus BX61 fluorescence microscope and processed by the software Volocity software from PerkinElmer.

#### Phospho-lipid binding assay

Purified GST-PH domain fusion proteins with concentration of 1 μg/mL were prepared in TBST buffer containing 2% of skim milk. PIP-strips were inoculated in the above solution for 16 hours at 4°C. The blots were subsequently processed following an anti-GST Far-Western protocol, and visualized by ECL.

## Authors' contributions

XYX, JJ and YYM designed the research. XYX performed the study. JJ contributed the experimental results. XYX, JJ and YYM analyzed the data and wrote the manuscript. All authors conceived the study and wrote, edited and approved the manuscript.

## Supplementary Material

Additional File 1 **supplementary results, tables and figures**. supplementary results, tables (Table S1, S2) and figures (Figure S1, S2, S3, S4, S5, S6, S7, S8) Click here for file

Additional File 2 **The survey information of domains in four kingdoms**. common domains shared by all genomes (Table S1) and specific domains found in each kingdom (Table S2, S3, S4). Click here for file

Additional File 3 **The dependence of clustering coefficient on node degree in the 77 domain bigram networks**. The log-log plots of clustering coefficient versus the degree of node in networks. Click here for file
