# Supplementary material for: Evolutionary versatility of eukaryotic protein domains revealed by their bigram networks
Source: BMC Evol Biol. 2011 Aug 18;11:242. doi: 10.1186/1471-2148-11-242 (PMC3167776; doi:10.1186/1471-2148-11-242)
Supplement: Additional File 1 — supplementary results, tables and figures. supplementary results, tables (Table S1, S2) and figures (Figure S1, S2, S3, S4, S5, S6, S7, S8) [file 1471-2148-11-242-S1.PDF]

# Supplementary information for

# Evolutionary versatility of Eukaryotic protein domains revealed by their bigram networks

Table of contents

Supplementary Results....2
Survey of superfamily domains in 77 completely sequenced genomes across four eukaryotic kingdoms....2
Supplementary Tables and Figures....3
Table S1....3
Table S2....5
Figure S1....7
Figure S2....9
Figure S3....11
Figure S4....132
Figure S5....13
Figure S6....154
Figure S7....165
Figure S8....16
References....17

# Supplementary Results

**Survey of superfamily domains in 77 completely sequenced genomes across four eukaryotic kingdoms**.

Seventy-seven fully-sequenced genomes across four eukaryotic kingdoms of fungi, protista, plantae and metazoan are found to contain 1235 encoded domain families (superfamily annotations – see Methods for details). The levels of domain sharing across the four kingdoms are shown in Figure S1A. A total of 771 domains are shared by four kingdoms (see Figure S1A) and in which 190 domains (refer to Table S1 in additional file 2, gray box in Figure S1B), primarily found in structural proteins or to participate in transcription/translation and metabolic activities, are shared by all genomes.

There are 131 superfamily domains encoded only by metazoa genomes, and 25 domains shared only between metazoa genomes and protista genomes (see additional file 2: Table S2). Among these 156 domains, the majority are known to participate in animal-specific processes such as immune response and cell signaling. One may speculate that the sharing of domains between metazoa and protista may, to some extent, be a result of lateral gene transfer during parasite-host co-evolution[1]. Similarly, plantae and protists also share 40 domains that do not belong to other kingdoms (refer to Table S3 in additional file 2). The sharing of these domains is primarily due to photoautotrophic protista species, such as *ya* and *tl*, which contain, for example, "Photosystem I subunit PsaD" and "Subunit III of photosystem I reaction centre, PsaF" domains for photosynthesis. Plants share six domains with plasmodium *pl* and *py* (marked with * in Table S3 in additional file 2). It might be due to the fact that plasmodium species have an essential organelle apicoplast which has the common ancestor with chloroplast[2]. There are 26 domains exclusive for protista, where each is shared by no more than three organisms (refer to Table S4 in additional file 2) – a phenomena possibly ascribed to the diversity of their hosting species and the simplicity of their genomes that facilitates rapid evolution.

# Supplementary Tables and Figures

**Table S1** Taxonomic information and the numbers of proteins and the percent of proteins with domain assigned for each species.

| Abbr. | Species | Kingdom;Phylum/Subphylum;Class | | | #Proteins with domain assigned | %Proteins with domain assigned |
|---|---|---|---|---|---|---|
| ol | Oryzias | Metazoa | Vertebrata | Actinopterygii | 17366 | 66 |
| gc | Gasterosteus | | | | 18695 | 68 |
| to | Takifugu | | | | 14528 | 66 |
| tn | Tetraodon | | | | 15283 | 55 |
| da | Danio | | | | 25642 | 71 |
| xn | Xenopus | | | Amphibia | 19590 | 69 |
| gg | Gallus | | | Aves | 10582 | 48 |
| mm | Mus | | | Mammalia | 20458 | 63 |
| ru | Macaca | | | | 23298 | 64 |
| hs | Homo | | | | 27380 | 63 |
| xp | Pan | | | | 20451 | 62 |
| dg | Canis | | | | 14237 | 56 |
| bv | Bos | | | | 14162 | 50 |
| rn | Rattus | | | | 22460 | 67 |
| op | Monodelphis | | | | 23568 | 72 |
| oh | Ornithorhynchus | | | | 17013 | 69 |
| is | Ciona | | Chordata | Ascidiacea | 11489 | 57 |
| c0 | Ciona | | | | 12090 | 60 |
| tu | Strongylocentrotus | | Echinodermata | Echinoidea | 26020 | 61 |
| dm | Drosophila | | Arthropoda | Insecta | 11317 | 59 |
| dd | Drosophila | | | | 11556 | 58 |
| do | Drosophila | | | | 5743 | 58 |
| ag | Anopheles | | | | 7758 | 57 |
| ax | Aedes | | | | 9758 | 58 |
| ai | Apis | | | | 15312 | 55 |
| om | Bombyx | | | | 8398 | 39 |
| cw | Caenorhabditis | | Nematoda | Secernentea | 9000 | 47 |
| cl | Caenorhabditis | | | | 13951 | 51 |
| ce | Caenorhabditis | | | | 11177 | 49 |
| cy | Chlamydomonas | Plantae | Chlorophyta | Chlorophyceae | 6998 | 46 |
| ou | Ostreococcus | | | Prasinophyceae | 4062 | 53 |
| mw | Medicago | | Magnoliophyta | Magnoliopsida | 9005 | 32 |
| pt | Populus | | | | 24531 | 54 |
| at | Arabidopsis | | | | 18626 | 58 |
| os | Oryza | | | Liliopsida | 30538 | 46 |

| | | | | | | |
|---|---|---|---|---|---|---|
| nd | Stagonospora | Fungi | Ascomycota | Pezizomycotina | 6532 | 39 |
| an | Aspergillus | | | | 5646 | 59 |
| ao | Aspergillus | | | | 5520 | 56 |
| re | Trichoderma | | | | 5489 | 55 |
| fg | Fusarium | | | | 6515 | 49 |
| gr | Magnaporthe | | | | 5274 | 47 |
| ns | Neurospora | | | | 4398 | 41 |
| yl | Yarrowia | | | Saccharomycotina | 3682 | 55 |
| al | Candida | | | | 3427 | 56 |
| gl | Candida | | | | 2983 | 57 |
| dh | Debaromyces | | | | 3667 | 53 |
| go | Ashbya | | | | 2765 | 59 |
| kl | Kluyveromyces | | | | 2977 | 56 |
| kw | Kluyveromyces | | | | 2932 | 56 |
| y1 | Saccharomyces | | | | 3375 | 28 |
| sc | Saccharomyces | | | | 3333 | 50 |
| xs | Saccharomyces | | | | 3346 | 50 |
| y6 | Saccharomyces | | | | 3411 | 33 |
| y8 | Saccharomyces | | | | 3301 | 31 |
| po | Schizosaccharomyces | | | Taphrinomycotina | 3039 | 61 |
| cf | Cryptococcus | | Basidiomycota | Hymenomycetes | 3413 | 58 |
| or | Coprinopsis | | | Agaricomycetes | 5698 | 42 |
| lo | Laccaria | | | | 6547 | 32 |
| fc | Phanerochaete | | | Hymenomycetes | 5380 | 54 |
| um | Ustilago | | | Ustilaginomycetes | 3626 | 56 |
| eu | Encephalitozoon | | Microspora | Unikaryonidae | 1014 | 51 |
| rm | Cryptosporidium | Protista | Apicomplexa | Conoidasida | 1584 | 40 |
| pl | Plasmodium | | | Aconoidasida | 2234 | 41 |
| py | Plasmodium | | | | 1996 | 31 |
| nu | Theileria | | | | 1675 | 44 |
| pv | Theileria | | | | 1696 | 42 |
| hy | Tetrahymena | | Ciliophora | Oligohymenophorea | 10509 | 38 |
| em | Leishmania | | Euglenozoa | Kinetoplastea | 3949 | 48 |
| tb | Trypanosoma | | | | 3637 | 42 |
| uz | Trypanosoma | | | | 8194 | 42 |
| en | Entamoeba | | Amoebozoa | Archamoebae | 4744 | 49 |
| dt | Dictyostelium | | | Mycetozoa | 6410 | 47 |
| tx | Trichomonas | | Metamonada | Parabasalia | 21960 | 22 |
| ya | Cyanidioschyzon | | Rhodophyta | Bangiophyceae | 3013 | 60 |
| tl | Thalassiosira | | Ochrophyta | Coscinodiscophyceae | 6420 | 56 |
| ra | Phytophthora | | Heterokontophyta | Oomycetes | 8527 | 53 |
| sj | Phytophthora | | | | 9381 | 49 |

**Table S2** Domains in the innermost cores of kingdom domain bigram networks**.** There are ten nested cores in metazoan (Me), six in plantae (Pl), eight in protista (Pr) and five in fungi (Fu). Column 3~6 show the domain networking versatilities of domains (sorted by column 3) which are in at least one innermost core of the network.

| Interpro ID | Superfamily | Me.(10) | Pl.(6) | Pr.(8) | Fu.(5) | Desc. |
|---|---|---|---|---|---|---|
| IPR002110* | Ankyrin repeat | 10 | 6 | 8 | 5 | Protein-protein interaction |
| IPR000225* | ARM repeat | 10 | 6 | 8 | 5 | General or several functions |
| IPR003405* | P-loop containing nucleoside triphosphate hydrolases | 10 | 6 | 8 | 5 | Small molecule binding |
| IPR004166* | Protein kinase-like (PK-like) | 10 | 6 | 8 | 5 | Kinases and phosphatases and inhibitors |
| IPR003613* | RING/U-box | 10 | 6 | 8 | 5 | DNA replication, recombination, repair |
| IPR001680* | WD40 repeat-like | 10 | 6 | 8 | 5 | General or several functions |
| IPR002048* | EF-hand | 10 | 6 | 8 | 4 | General or several functions |
| IPR000494* | L domain-like | 10 | 6 | 8 | 4 | General or several functions |
| IPR003590* | RNI-like | 10 | 6 | 8 | 4 | Cell adhesion |
| IPR011991* | Winged helix DNA-binding domain | 10 | 6 | 7 | 5 | DNA-binding |
| IPR001098* | DNA/RNA polymerases | 10 | 6 | 5 | 4 | DNA replication, recombination, repair |
| IPR000504* | RNA-binding domain, RBD | 10 | 6 | 5 | 4 | RNA processing and modification |
| IPR011011* | FYVE/PHD zinc finger | 10 | 5 | 8 | 4 | DNA replication, recombination, repair |
| IPR015496 | Ubiquitin-like | 10 | 5 | 6 | 4 | General or several functions |
| IPR002035 | vWA-like | 10 | 5 | 6 | 3 | Cell adhesion |
| IPR008185 | DNase I-like | 10 | 5 | 5 | 4 | DNA replication, recombination, repair |
| IPR001849* | PH domain-like | 10 | 4 | 8 | 4 | Signal transduction |
| IPR013069 | POZ domain | 10 | 4 | 6 | 2 | Protein-protein interaction |
| IPR004124 | Concanavalin A-like lectins/glucanases | 10 | 4 | 5 | 4 | Secondary metabolism |
| IPR008979 | Galactose-binding domain-like | 10 | 4 | 5 | 4 | Carbohydrate transport and metabolism |
| IPR006025 | Metalloproteases (zincins), catalytic domain | 10 | 4 | 3 | 4 | Proteases |
| IPR001478 | PDZ domain-like | 10 | 3 | 6 | 1 | Signal transduction |
| IPR013017 | NHL repeat | 10 | 3 | 4 | 3 | Other enzymes |
| IPR009003 | Trypsin-like serine proteases | 10 | 3 | 4 | 5 | Proteases |
| IPR001190 | SRCR-like | 10 | 3 | 1 | - | Receptor activity |
| IPR003118 | SAM/Pointed domain | 10 | 2 | 7 | 2 | DNA-binding |
| IPR006209 | EGF/Laminin | 10 | 2 | 2 | - | Cell adhesion |
| IPR000538 | C-type lectin-like | 10 | 2 | - | - | Cell adhesion |
| IPR001452 | SH3-domain | 10 | 1 | 4 | 3 | Signal transduction |
| IPR000276 | Family A G protein-coupled receptor-like | 10 | 1 | 3 | 1 | Signal transduction |
| IPR003961 | Fibronectin type III | 10 | 1 | 3 | 1 | Cell adhesion |
| IPR009030 | Growth factor receptor domain | 10 | - | 4 | - | Signal transduction |
| IPR007110 | Immunoglobulin | 10 | - | 1 | - | Cell adhesion |
| IPR013806 | Kringle-like | 10 | - | 1 | - | Blood clotting |
| IPR002172 | LDL receptor-like module | 10 | - | 1 | - | Transport, plasma system |
| IPR000884 | TSP-1 type 1 repeat | 10 | - | 1 | - | Cell adhesion |
| IPR000436 | Complement control module/SCR domain | 10 | - | - | - | Immune response |
| IPR000488 | DEATH domain | 10 | - | - | - | Cell cycle, Apoptosis |
| IPR000859 | Spermadhesin, CUB domain | 10 | - | - | - | General or several functions |
| IPR001440* | TPR-like | 9 | 6 | 8 | 5 | Protein-protein interaction |

| | | | | | | |
|---|---|---|---|---|---|---|
| IPR008973 | C2 domain (Calcium/lipid-binding domain, CaLB) | 9 | 4 | 8 | 4 | Signal transduction |
| IPR011044 | YVTN repeat-like/Quinoprotein amine dehydrogenase | 9 | 4 | 4 | 5 | Other enzymes |
| IPR012337 | Ribonuclease H-like | 8 | 6 | 5 | 4 | Nucleotide transport and metabolism |
| IPR012336 | Thioredoxin-like | 8 | 6 | 4 | 4 | Oxidation/Reduction |
| IPR000340 | (Phosphotyrosine protein) phosphatases II | 8 | 3 | 8 | 3 | Kinases and phosphatases and inhibitors |
| IPR012393 | Tricorn protease domain 2 | 8 | 1 | 4 | 5 | Proteases |
| IPR001810* | F-box domain | 7 | 6 | 7 | 5 | Protein-protein interaction |
| IPR002941* | S-adenosyl-L-methionine-dependent methyltransferases | 7 | 6 | 6 | 5 | Transferases |
| IPR000173 | NAD(P)-binding Rossmann-fold domains | 7 | 4 | 5 | 5 | Small molecule binding |
| IPR013027 | FAD/NAD(P)-binding domain | 7 | 4 | 4 | 5 | Small molecule binding |
| IPR003089* | alpha/beta-Hydrolases | 6 | 4 | 8 | 5 | Other enzymes |
| IPR002469 | TolB, C-terminal domain | 6 | 4 | 3 | 5 | Proteases |
| IPR001128 | Cytochrome P450 | 6 | 2 | 1 | 5 | Oxidation/Reduction |

*Domains which are found to be in at least two innermost cores of the networks.

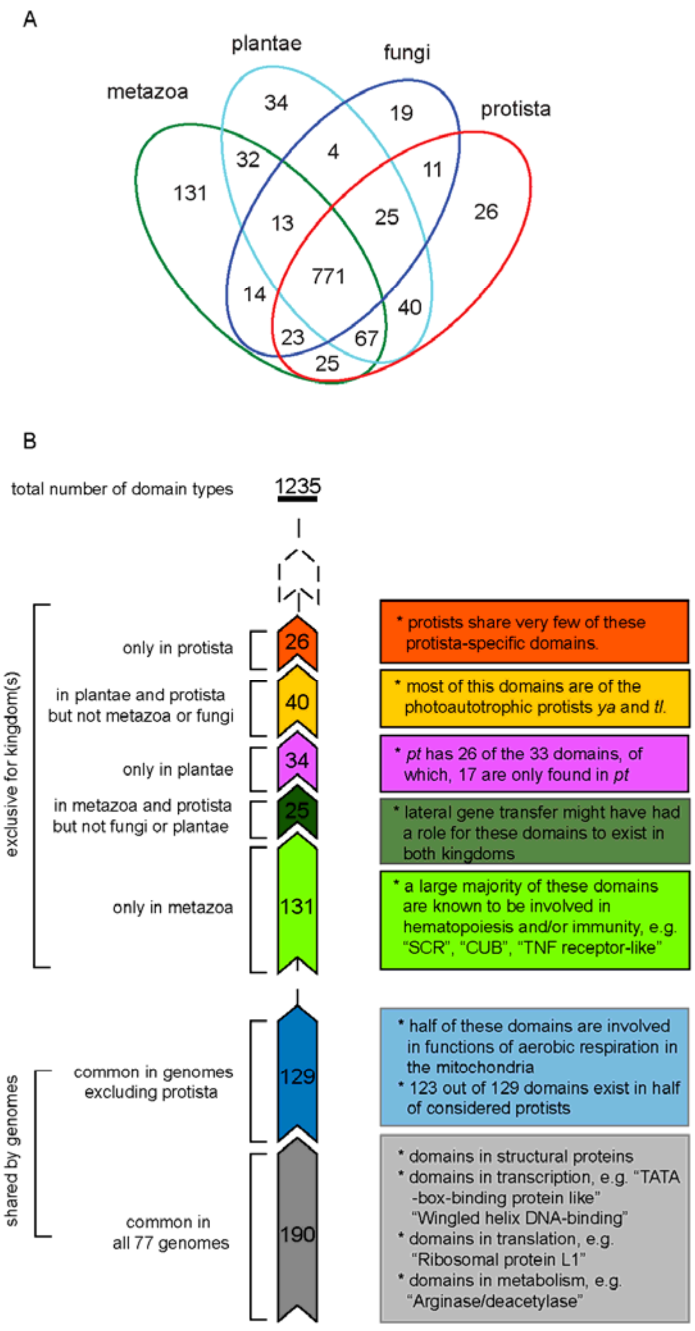


**Figure S1** Survey of superfamily domains in 77 completely sequenced genomes across four eukaryotic kingdoms.

There are 1235 domains in total assigned to proteins of 77 eukaryotic genomes. The

levels of domain sharing across the four kingdoms are shown in panel A. One hundred and ninety domain types (about 15%) are shared by all organisms, most of which carry basic and essential functions (described in the gray box in B).

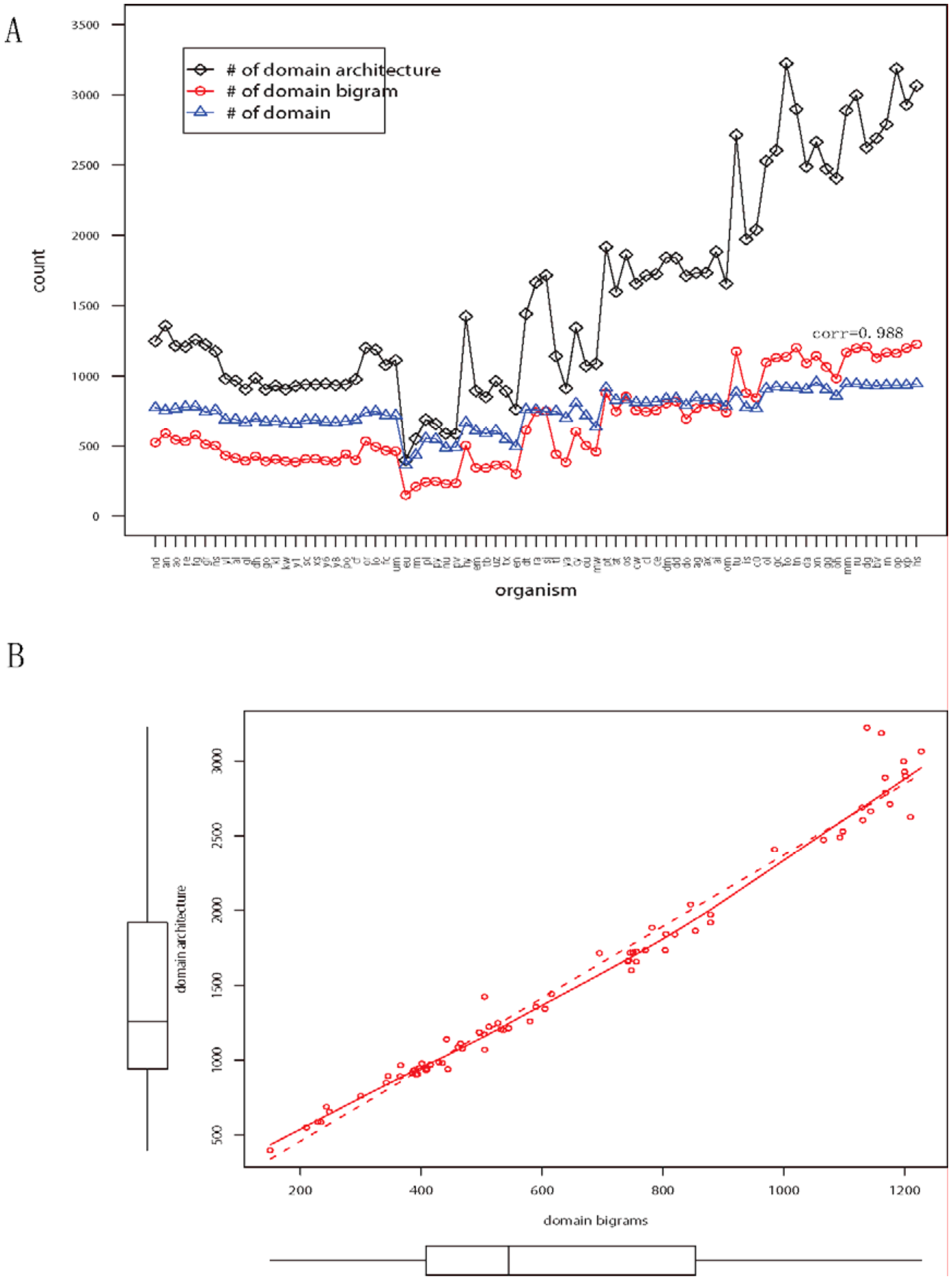


**Figure S2** The number of distinct domains, domain bigrams and domain architectures across analyzed genomes.

**A:** Along *x*-axis the eukaryotic organisms are ordered in increasing organism complexity. The number of bigrams and the number of domain architectures tend to

increase with genome complexity whereas the number of domain types stays relatively constant across genomes. This suggests that both bigrams and domain architectures can be viewed as silhouettes of biological complexity. B: The figure shows the number of domain bigrams and the numbers of domain architectures are highly correlated with correlation coefficient 0.988. The mean and standard deviation of bigram numbers and those of domain-architecture numbers are also shown.

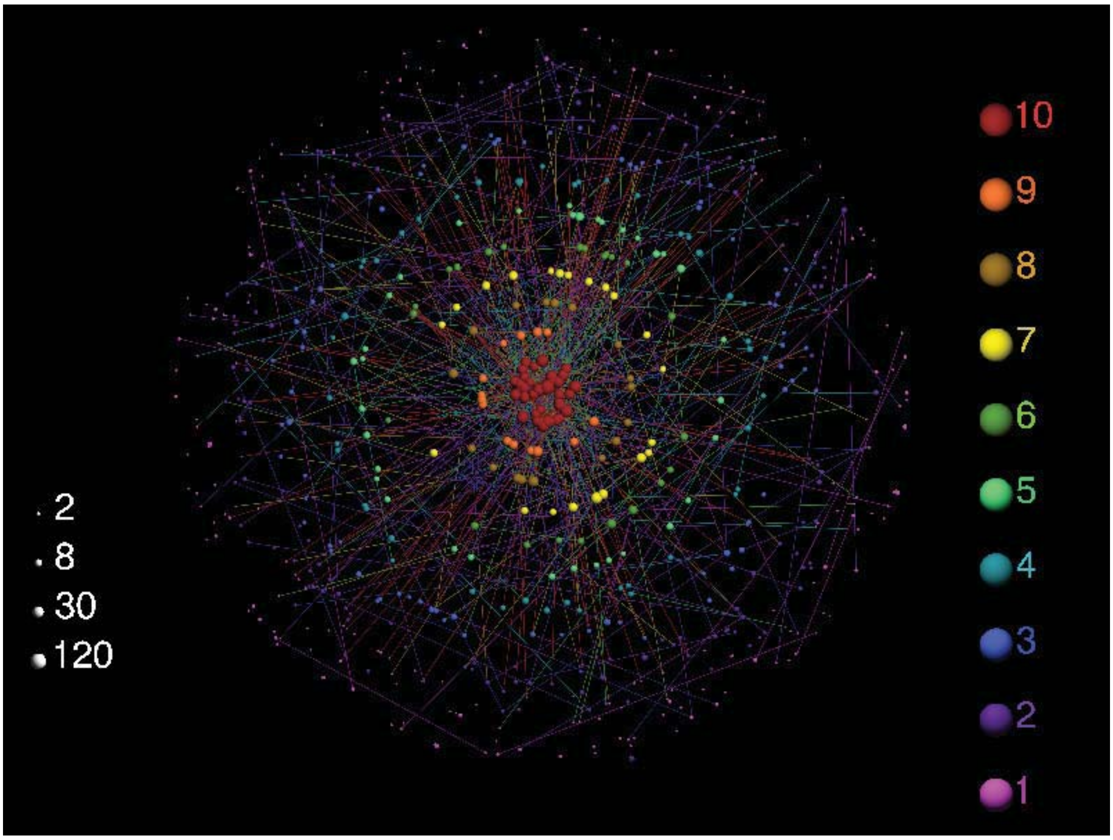


**Figure S3 Visualization of metazoan domain bigram network.**

For simple illustrative purpose, this plot was generated by the LArge NETwork VIsualization tool:http://xavier.informatics.indiana.edu/lanet-vi/. Nodes of the network were ordered by their networking versatilities. The size of the node represents the value of degree (white nodes listed in left side) and the color of the node codes the different levels of k-core (colorful nodes listed in the right side)

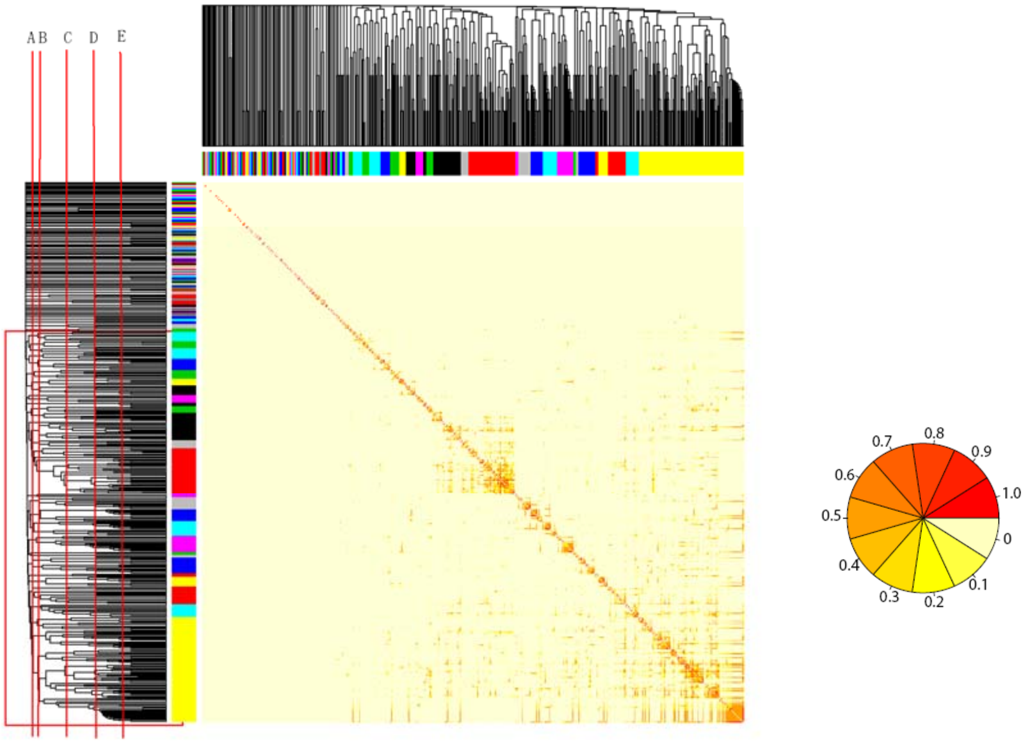


**Figure S4 The topological overlap matrix corresponding to human domain bigram network.**

The hierarchical clustering dendrogram with color bar representing the ordered module assignments quantifies the relation between the different modules. The color code of the matrix denotes the degree of topological overlap shown in the matrix (see the right color pie). Five red vertical lines across column dendrogram show five different cutoff levels used in Figure 4 at 0.95 for line A, 0.9 for line B, 0.7 for line C, 0.5 for line D and 0.3 for line E.

α-**Actin**

```
hActin          MCEEEDSTALVCDNGSGLCKAGFAGDDAPRAVFPSIVGRPRHQGVMVGMGQKDSYVGDEA 60
scActin         --MDSEVAALVIDNGSGMCKAGFAGDDAPRAVFPSIVGRPRHQGIMVGMGQKDSYVGDEA 58
                   :.: :*** *****:**************************:***************

hActin          QSKRGILTLKYPIEHGIITNWDDMEKIWHHSFYNELRVAPEEHPTLLTEAPLNPKANREK 120
scActin         QSKRGILTLRYPIEHGIVTNWDDMEKIWHHTFYNELRVAPEEHPVLLTEAPMNPKSNREK 118
                *********:*******:************:*************.******:***:****

hActin          MTQIMFETFNVPAMYVAIQAVLSLYASGRTTGIVLDSGDGVTHNVPIYEGYALPHAIMRL 180
scActin         MTQIMFETFNVPAFYVSIQAVLSLYSSGRTTGIVLDSGDGVTHVVPIYAGFSLPHAILRI 178
                *************:**:********:***************** **** *::*****:*:

hActin          DLAGRDLTDYLMKILTERGYSFVTTAEREIVRDIKEKLCYVALDFENEMATAASSSSLEK 240
scActin         DLAGRDLTDYLMKILSERGYSFSTTAEREIVRDIKEKLCYVALDFEQEMQTAAQSSSIEK 238
                ***************:****** ***********************:** ***.***:**

hActin          SYELPDGQVITIGNERFRCPETLFQPSFIGMESAGIHETTYNSIMKCDIDIRKDLYANNV 300
scActin         SYELPDGQVITIGNERFRAPEALFHPSVLGLESAGIDQTTYNSIMKCDVDVRKELYGNIV 298
                ******************.**:**:**.:*:*****.:**********:*:**:**.* *

hActin          LSGGTTMYPGIADRMQKEITALAPSTMKIKIIAPPERKYSVWIGGSILASLSTFQQMWIS 360
scActin         MSGGTTMFPGIAERMQKEITALAPSSMKVKIIAPPERKYSVWIGGSILASLTTFQQMWIS 358
                :******:****:************:**:**********************:********

hActin          KQEYDEAGPSIVHRKCF 377
scActin         KQEYDESGPSIVHHKCF 375
                ******:******:***
```

**Histone H2B**

```
hH2B            MLRTEVPRLPRSTTAIVWSCHLMATASAMAGPSSETTSEEQLITQEPKEANSTTSQKQSK 60
scH2B/HTB1      -----------------------MSAKAEKKPASKAPAEKKPAAK--KTSTSTDGKKRSK 35
                                        :*.*   *:*::.:*::  ::  * :.** .:*:**

hH2B            QRKRGRHGPRRCHSNCRGDSFATYFRRVLKQVHQGLSLSREAVSVMDSLVHDILDRIATE 120
scH2B/HTB1      ARK---------------ETYSSYIYKVLKQTHPDTGISQKSMSILNSFVNDIFERIATE 80
                 **               :::::*: :****.* . .:*::::*:::*:*:**::*****

hH2B            AGRLARSTKRQTITAWETRMAVRLLLPGQMGKLAESEGTKAVLRTSLYAIQQQRK 175
scH2B/HTB1      ASKLAAYNKKSTISAREIQTAVRLILPGELAKHAVSEGTRAVTK---YSSSTQA- 131
                *.:**  .*:.**:* * : ****:***::.* * ****:** :   *: . *
```

**Figure S5** Sequence alignments between yeast and human α-Actin (scActin and hsActin respectively), and between yeast and human Histone H2B (scH2B/HTB1 and hsH2B respectively).

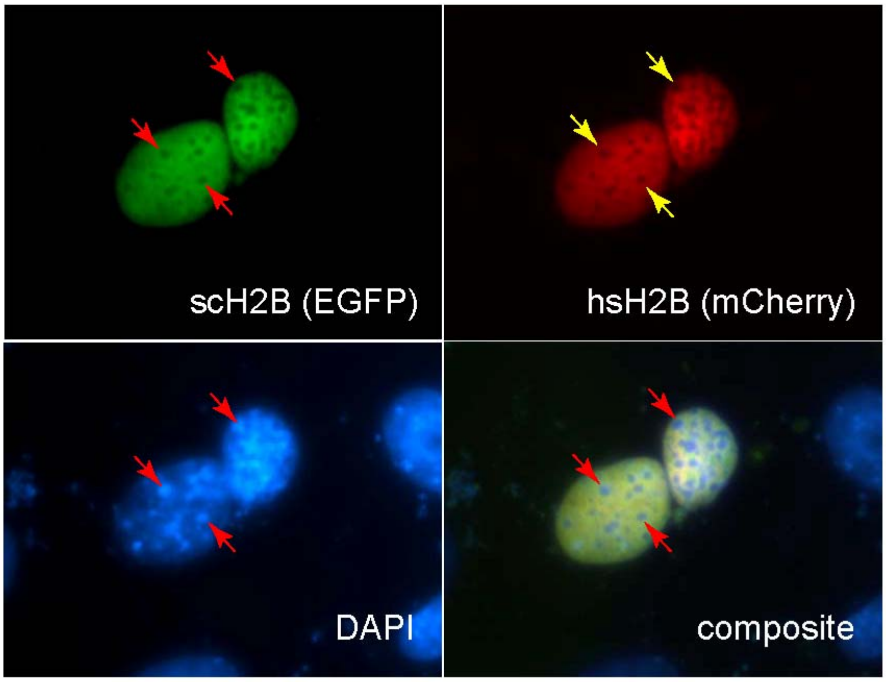


**Figure S6** A complete co-localization of yeast and human H2B proteins/domains in the nucleus of NIH-3T3 cells.

Yeast and human H2B sequences (scH2B and hsH2B respectively) are co-expressed as EGFP- and mCherry-fusion proteins respectively in NIH-3T3 cells. Consistent with the observation in Figure 6B, scH2B and hsH2B expressions are seen only in the nucleus. Although scH2B2 and hsH2B were localized indistinguishably from each other, they were presented more prominently in nuclear areas that were weakly stained with DAPI (these areas are also known as the interchromatin space -- pointed by arrows and also shown in composite, lower right).

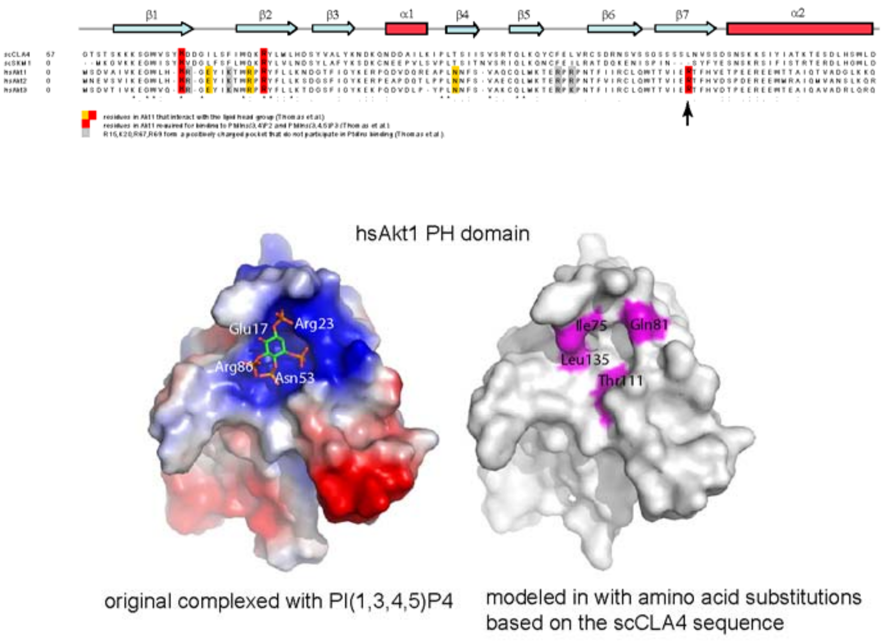


**Figure S7** Comparison of the amino acid sequences of PH domains in human Akt1 and yeast Cla4.

Upper panel: Five PH domain sequences (include those of the human Akt1-3 and of the yeast Cla4 and its homologue Skm1 protein) are aligned. Above the sequences is a scheme of secondary structure of the domain based on the *x*-ray crystallography structure of the PH domain of human Akt1 [3]. Residues surrounding the ligand-binding pocket are highlighted (in red and yellow). Residues highlighted in red have been shown to be critical for ligand binding (in the case of Akt1). Note that R86 of Akt1 that is required for PtdIns(3,4,5)P3 binding is not a conserved residue in the yeast PH domains (arrow). Bottom-left: *x*-ray crystallography structure of Akt PH domains binding to PtdIns(1,3,4,5)P4 (left). On the structure, dark blue marks surface with positive electric charge that forms the phospho-lipid-binding pocket. To its right: a schematic structure of the domain as is modeled with E17I, R23Q, N53T, R86L substitutions (right panel, now marked in purple) with residues found at equivalent positions on yeast Cla4. Additionally, it is interesting to note that residues R15,K20,R67,R69 in human Akt1 (together with the corresponding residues in Akt2/3 highlighted in gray) form a positively charged pocket that do not participate in PtdIns binding[3]. Although the function of this prominent pocket remains uncharacterized, it is

intriguing to notice the counterparts of these residues are completely absent from yeast PH domains in Cla4 and Skm1.

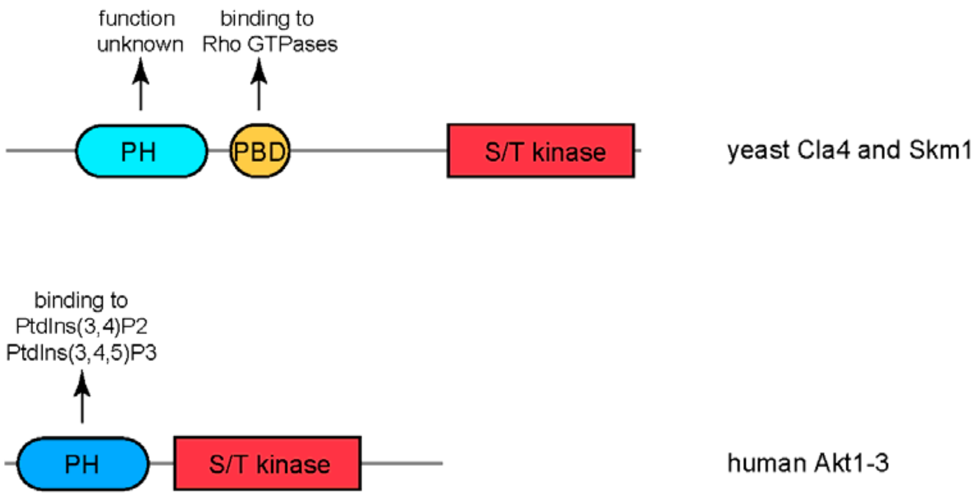


**Figure S8** Domain architectures of yeast Cla4 and human Akt family serine/threonine kinases.

# References


1. **Tordai H, Nagy A, Farkas K, Banyai L, Patthy L: Modules, multidomain proteins and organismic complexity. *FEBS J* 2005, 272(19):5064-5078.**
2. **Marechal E, Cesbron-Delauw M-F: The apicoplast: a new member of the plastid family. *Biology of the Cell* 2001, 6(5):200-205.**
3. **Thomas CC, Deak M, Alessi DR, van Aalten DM: High-resolution structure of the pleckstrin homology domain of protein kinase b/akt bound to phosphatidylinositol (3,4,5)-trisphosphate. *Current Biology* 2002, 12(14):1256-1262.**
