# Supplementary figures and images for: Evolutionary versatility of eukaryotic protein domains revealed by their bigram networks
Source: BMC Evol Biol. 2011 Aug 18;11:242. doi: 10.1186/1471-2148-11-242 (PMC3167776; doi:10.1186/1471-2148-11-242)

nd

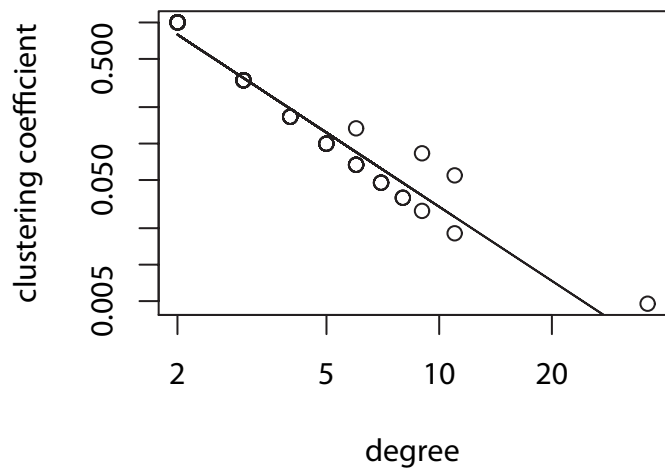

an

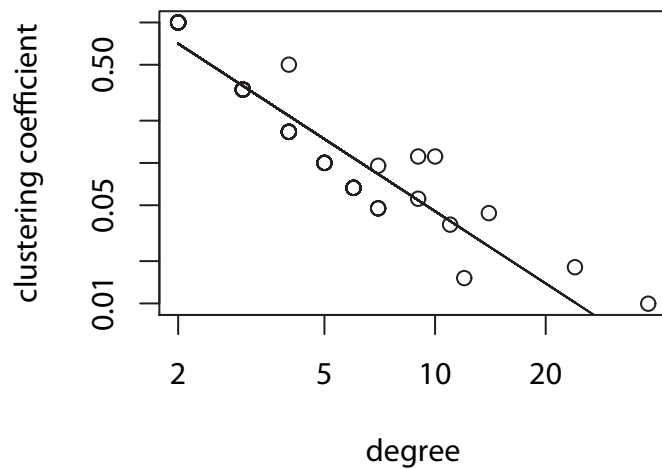

ao

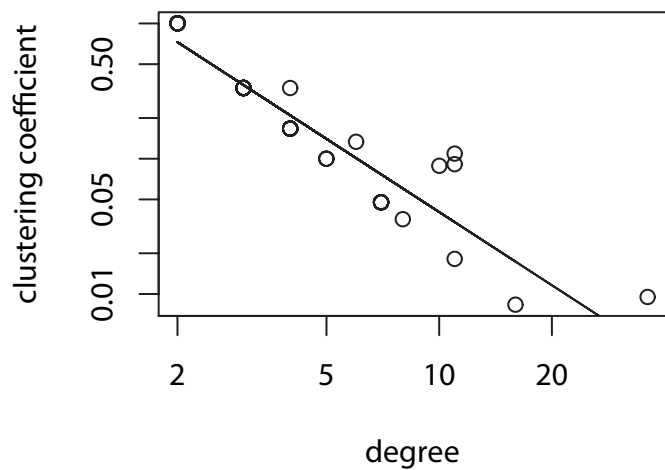

re

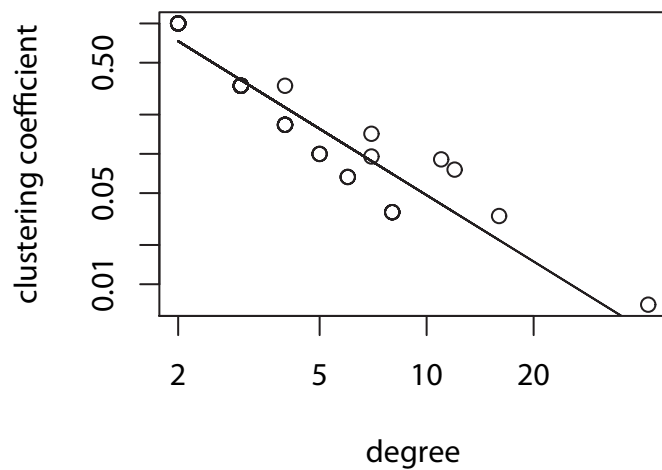

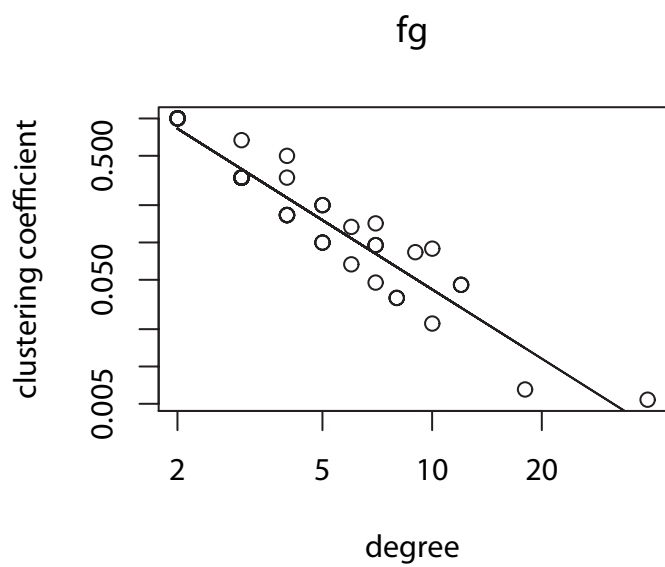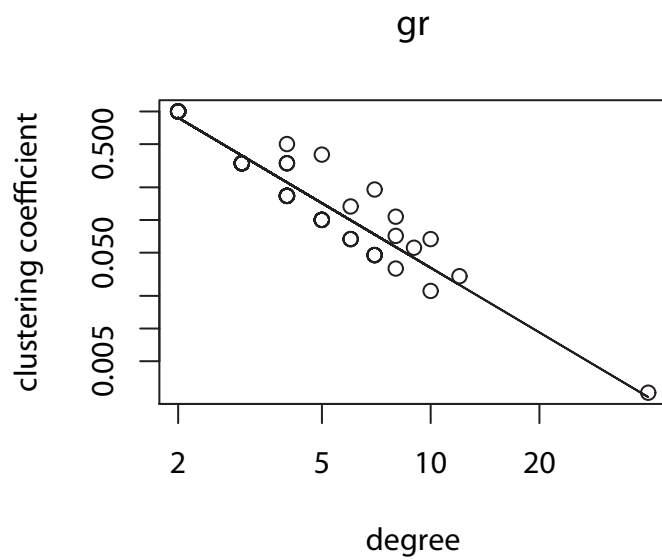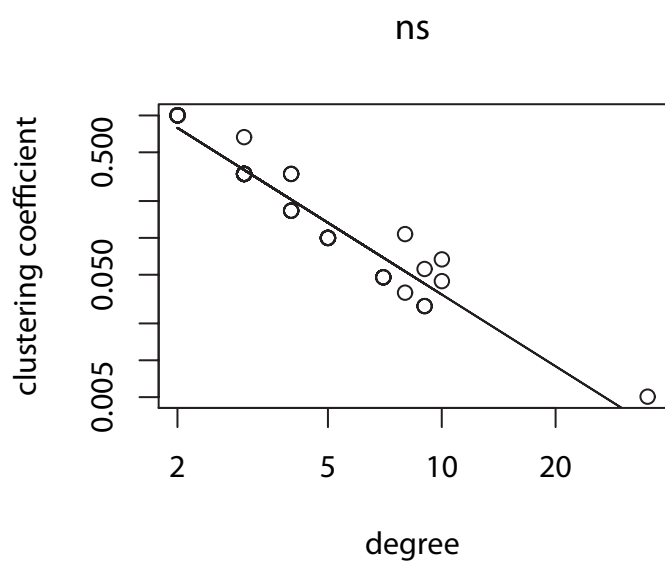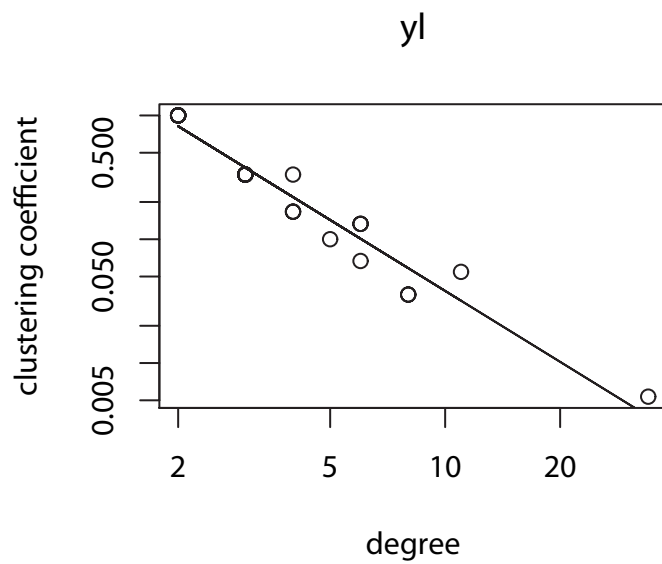

al

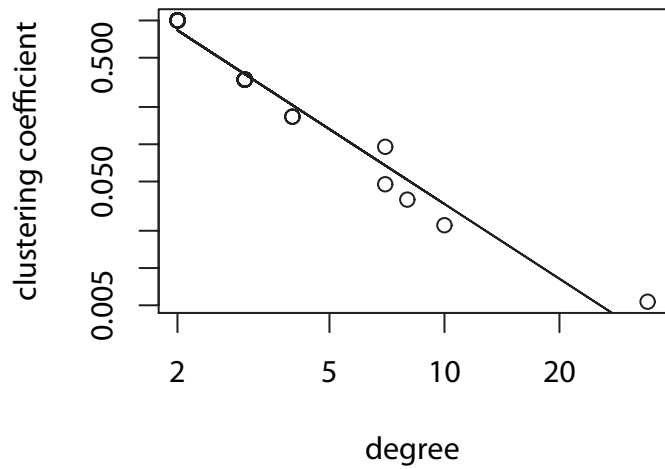

gl

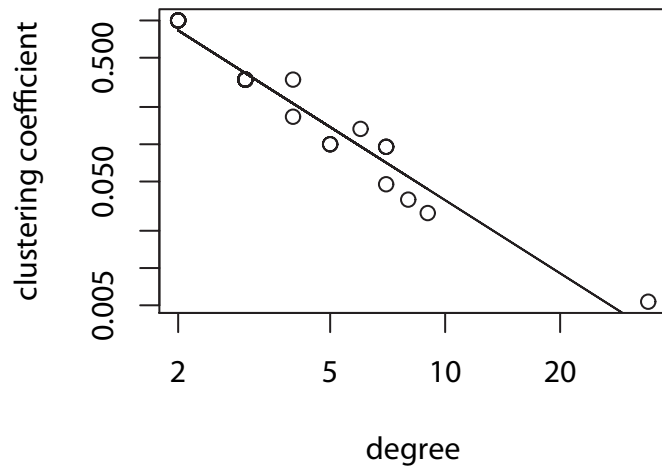

dh

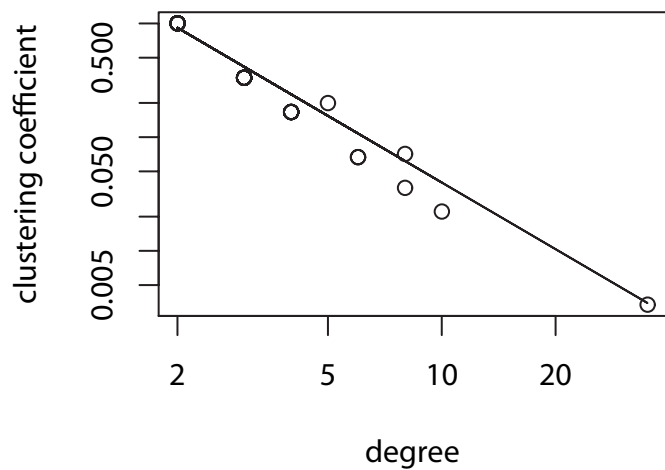

go

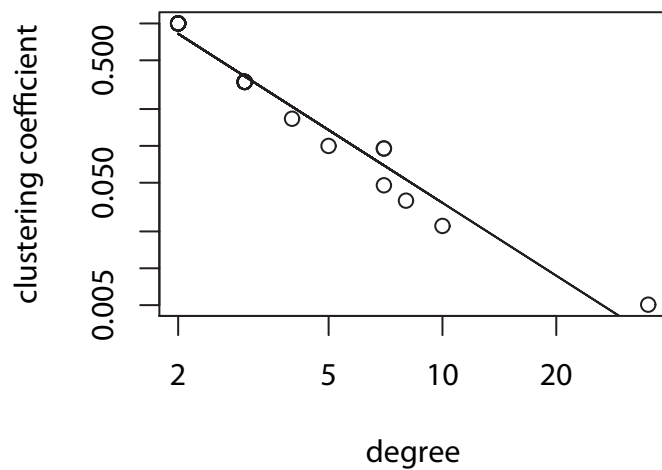

kl

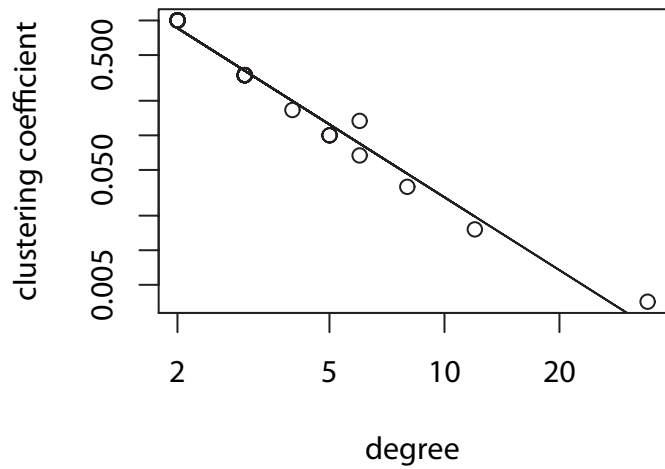

kw

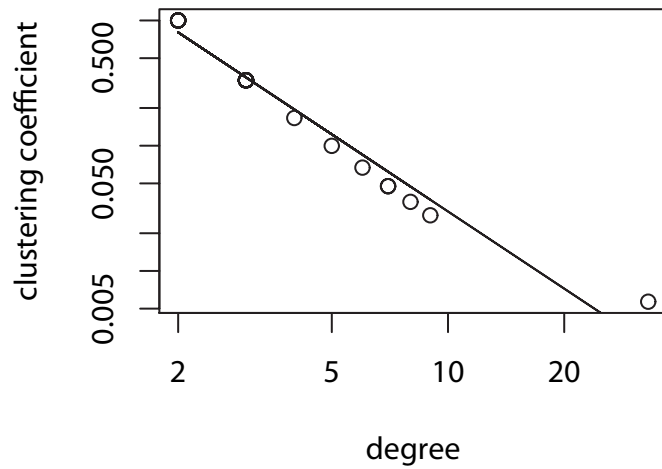

y1

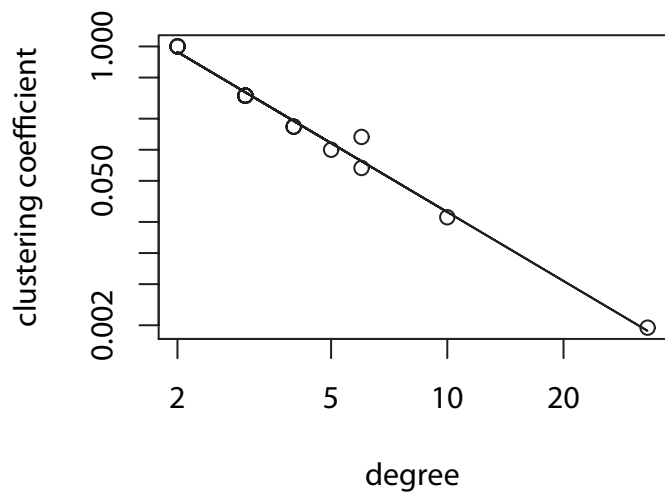

sc

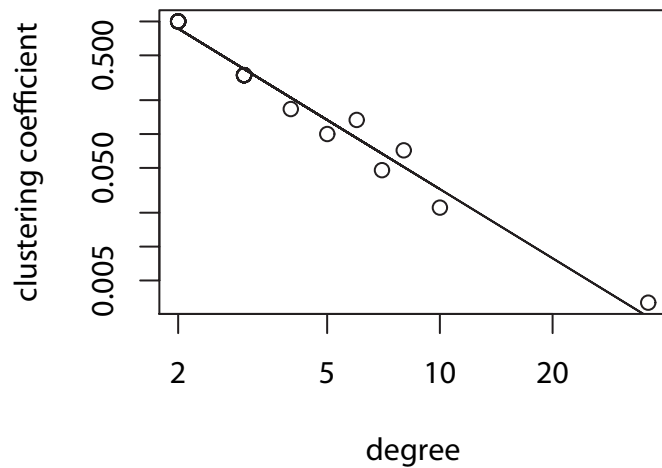

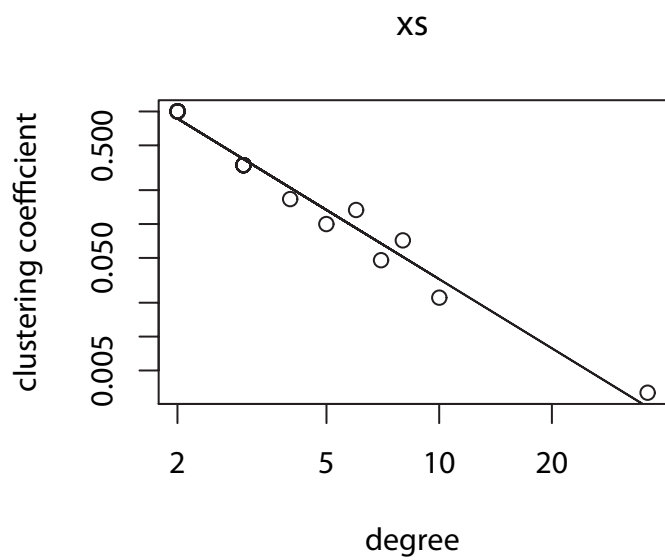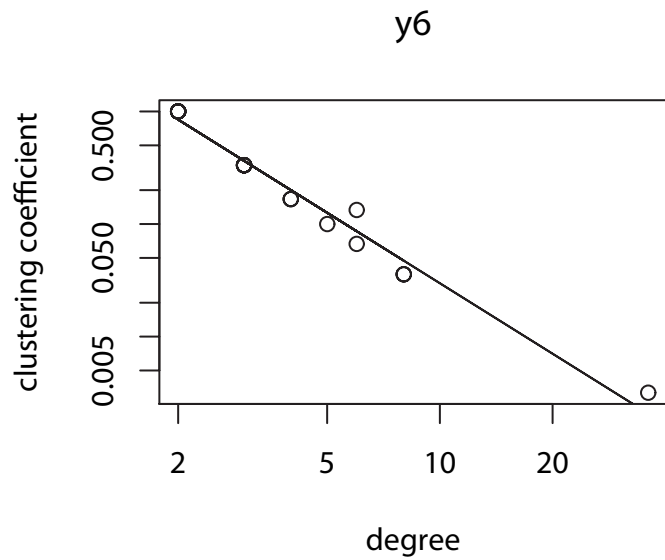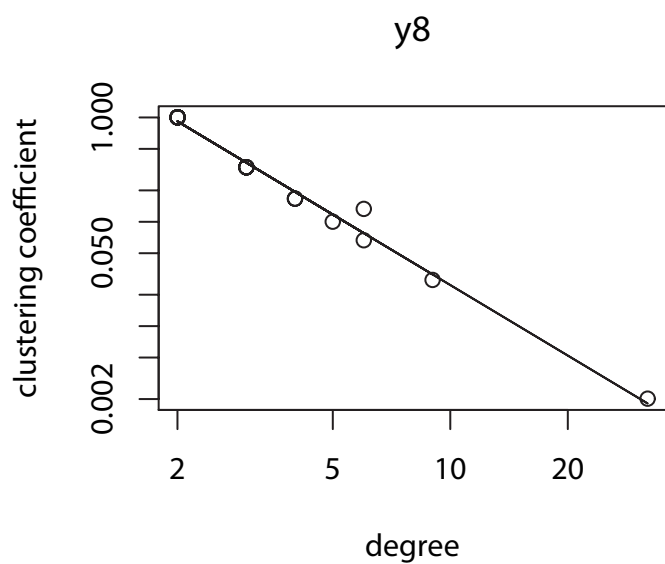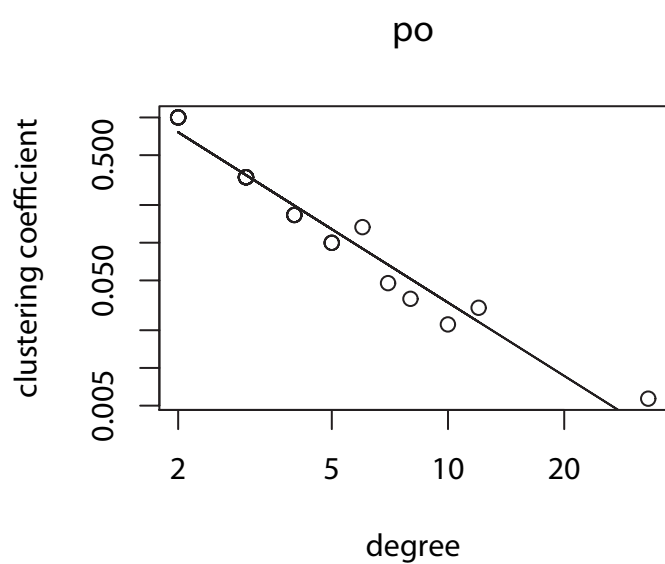

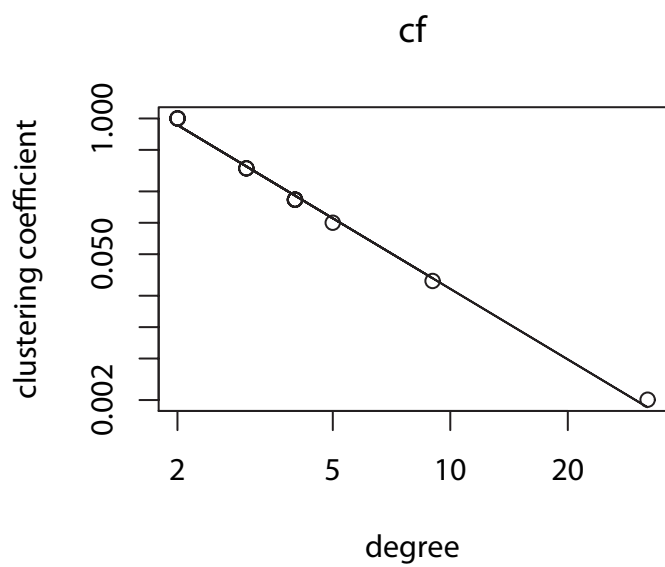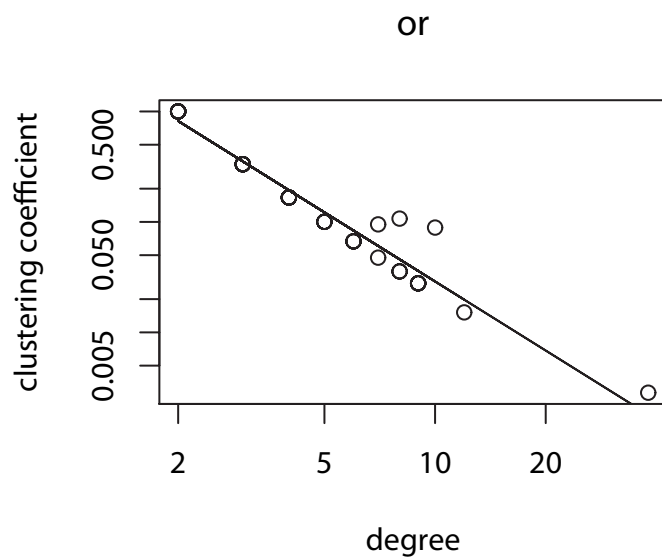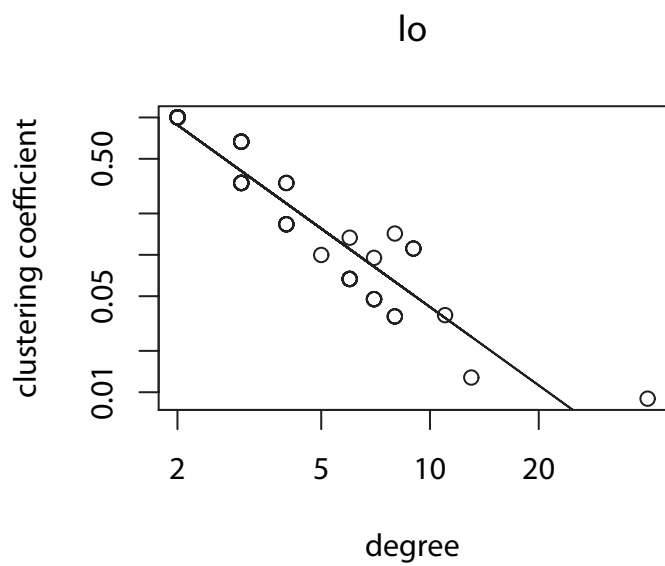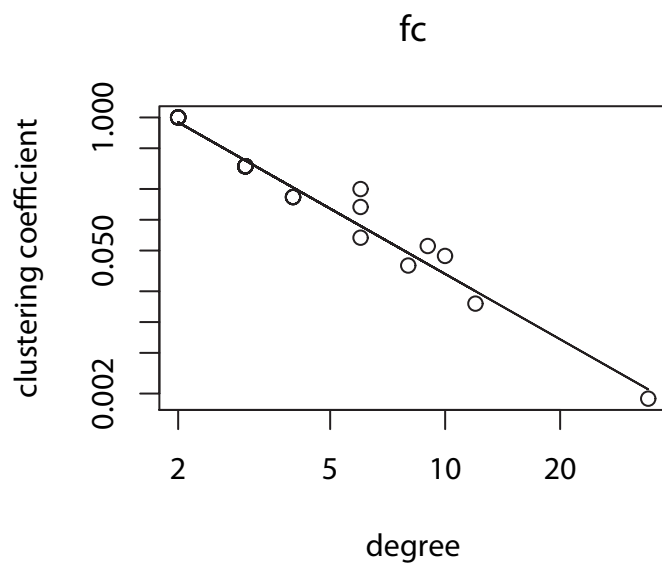

um

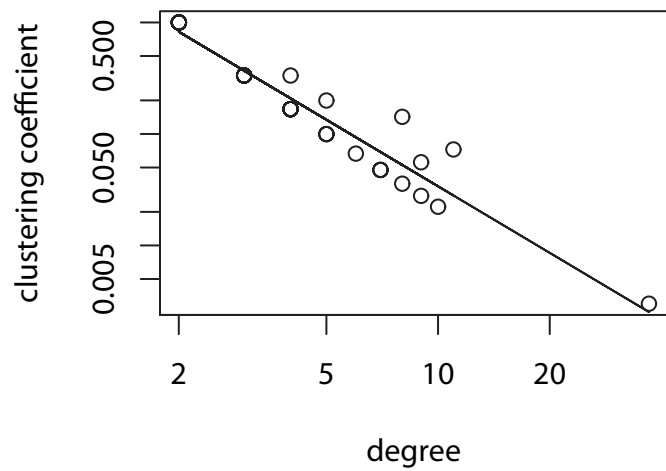

eu

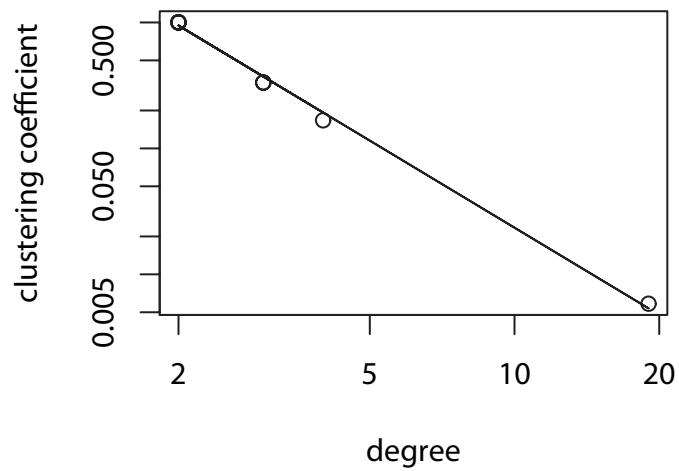

rm

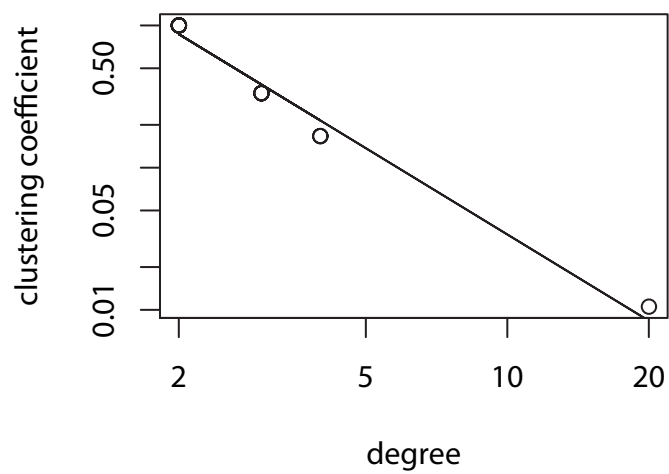

pl

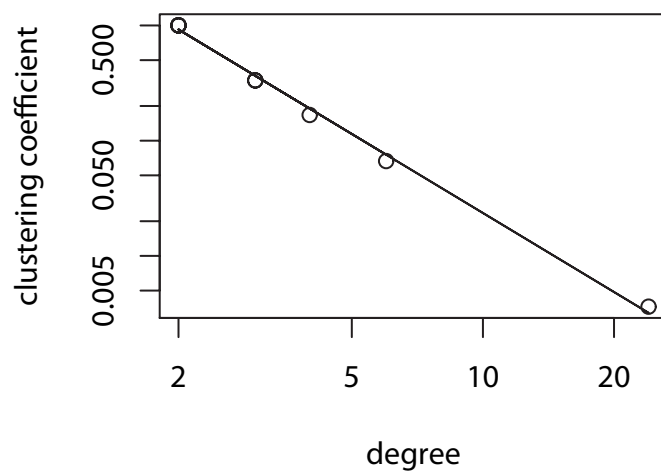

py

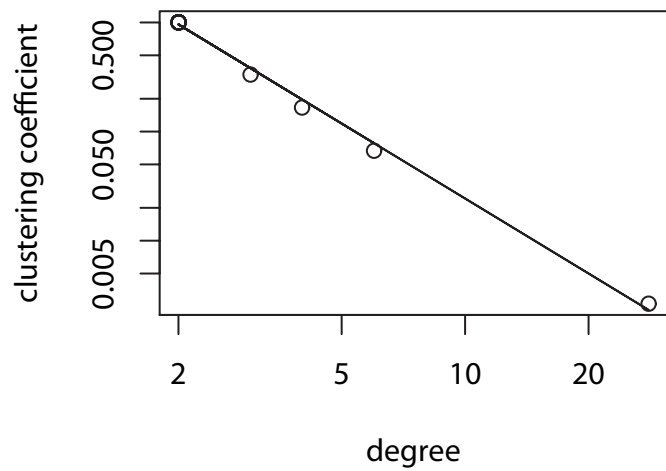

nu

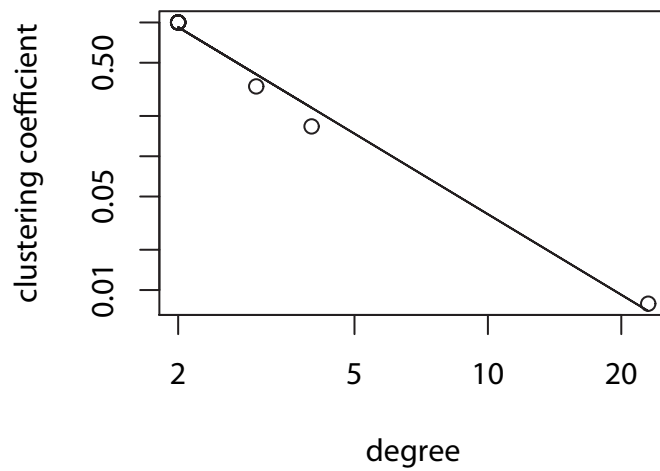

pv

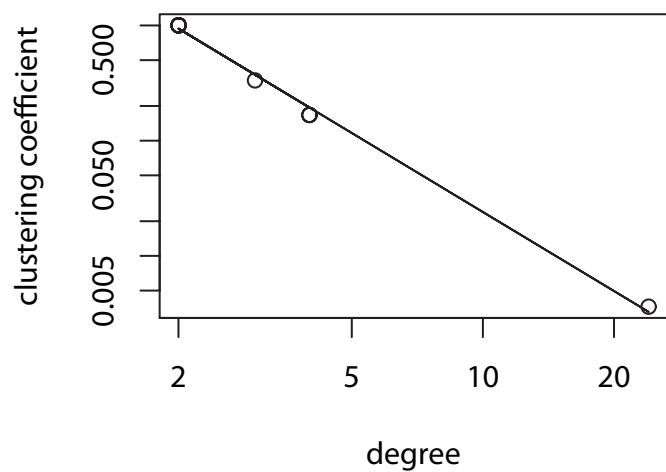

hy

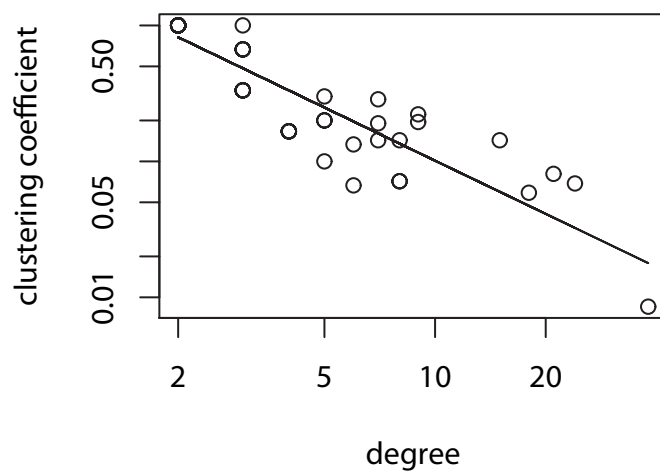

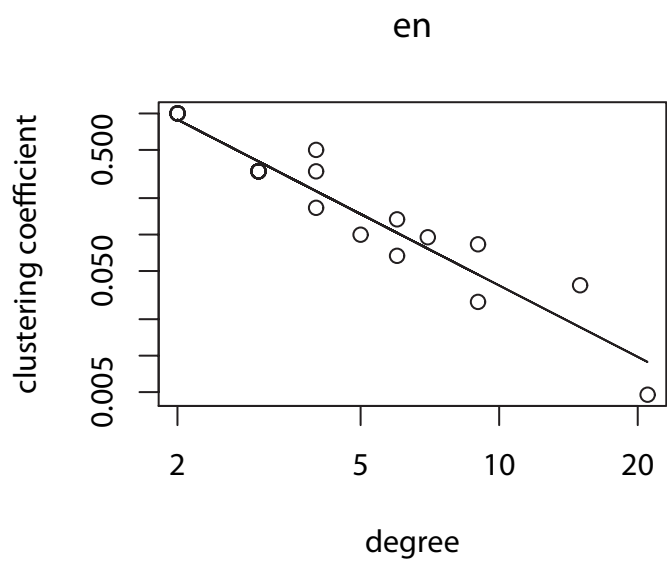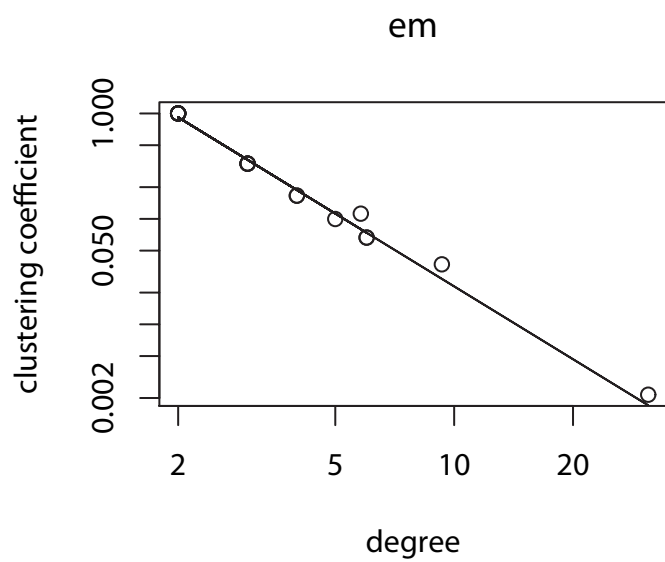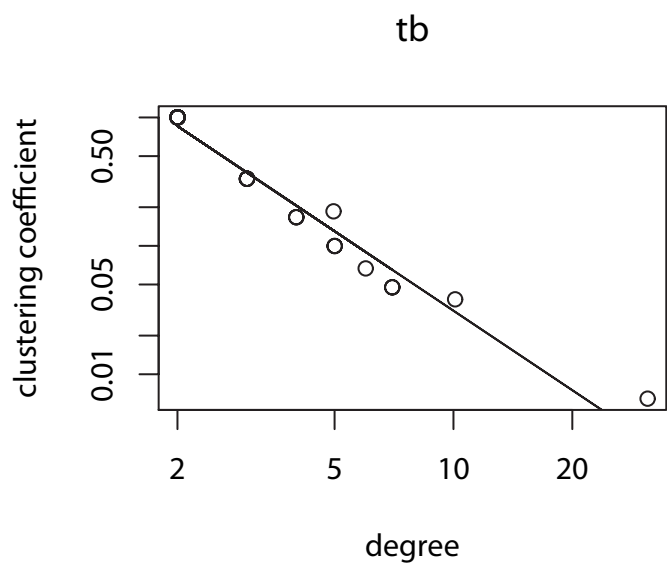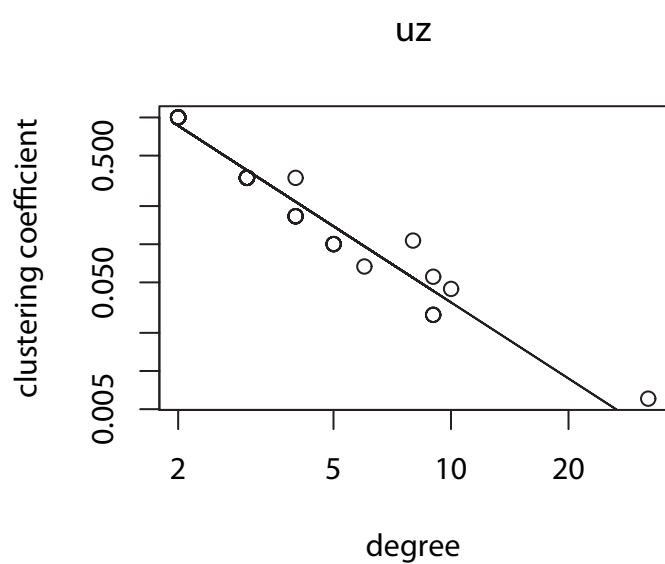

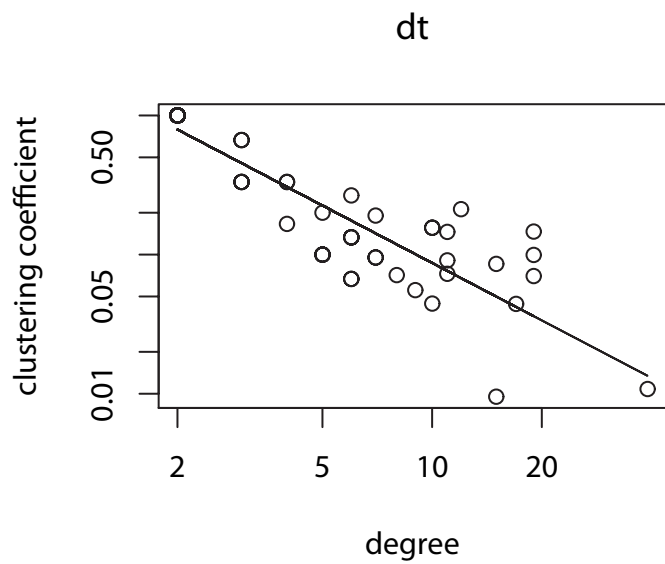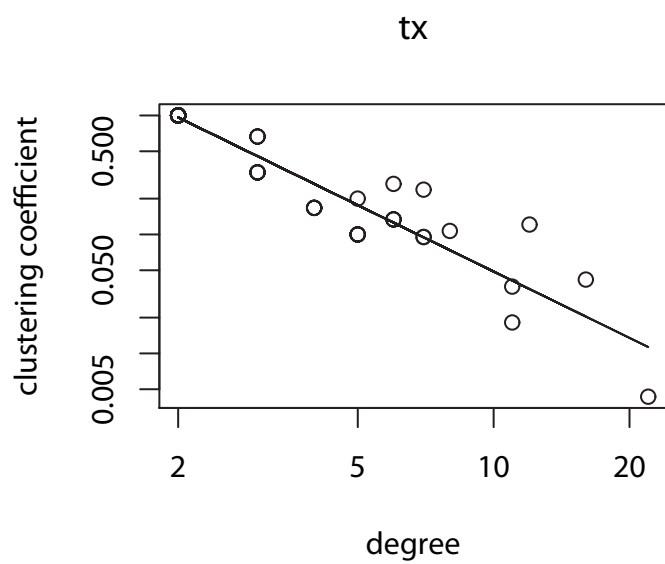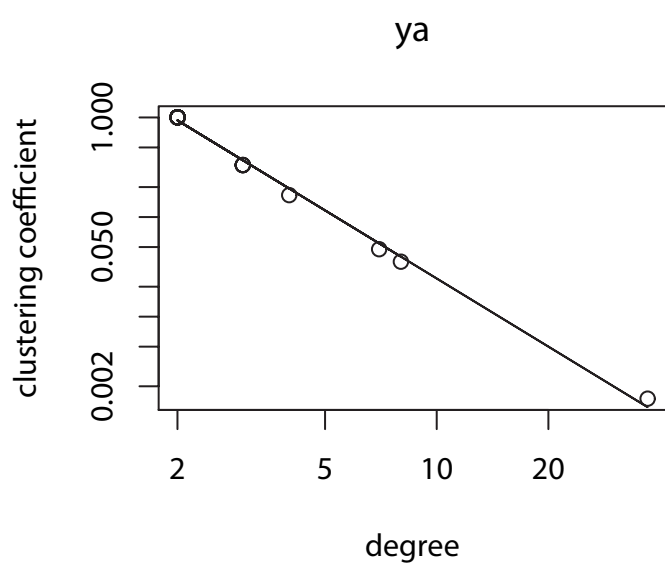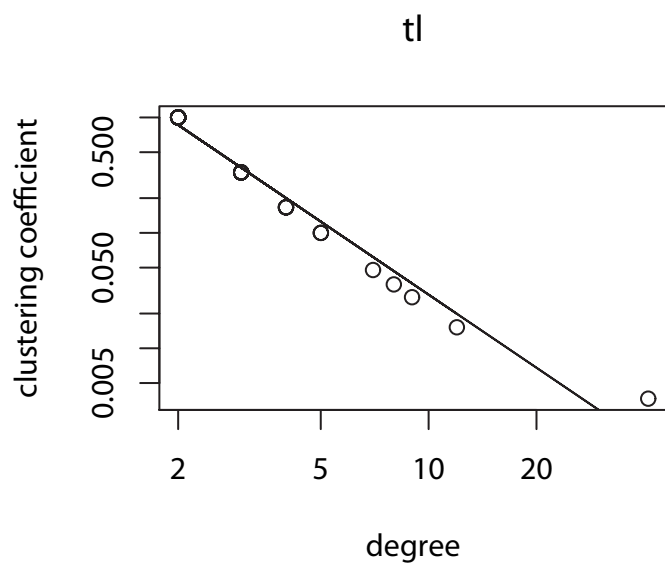

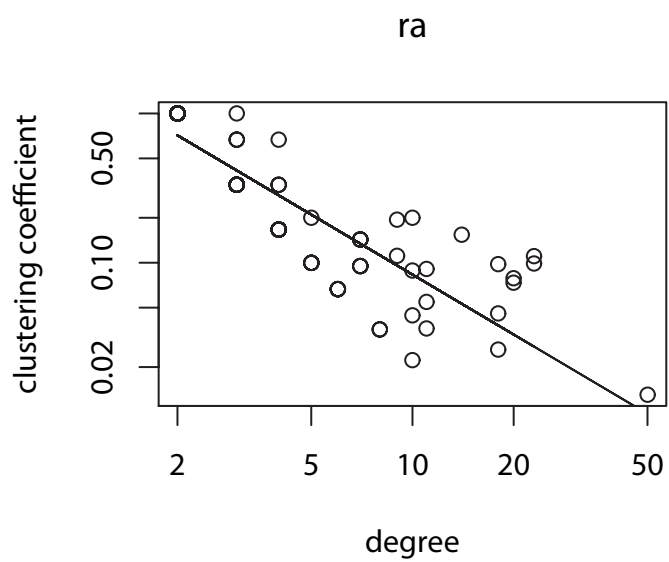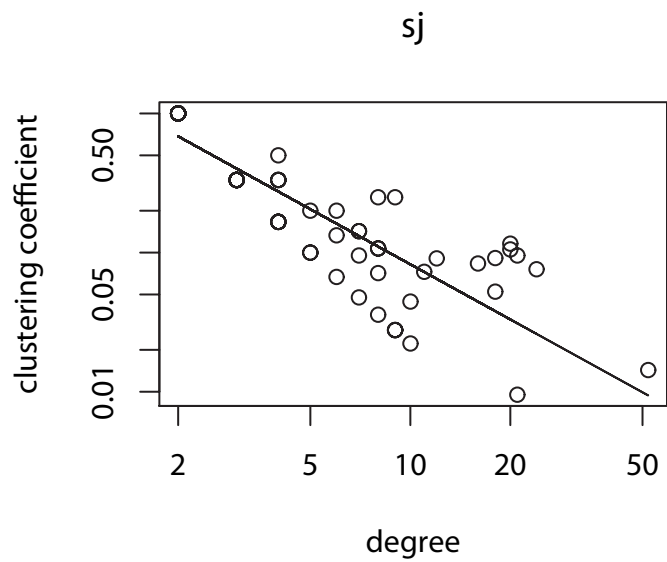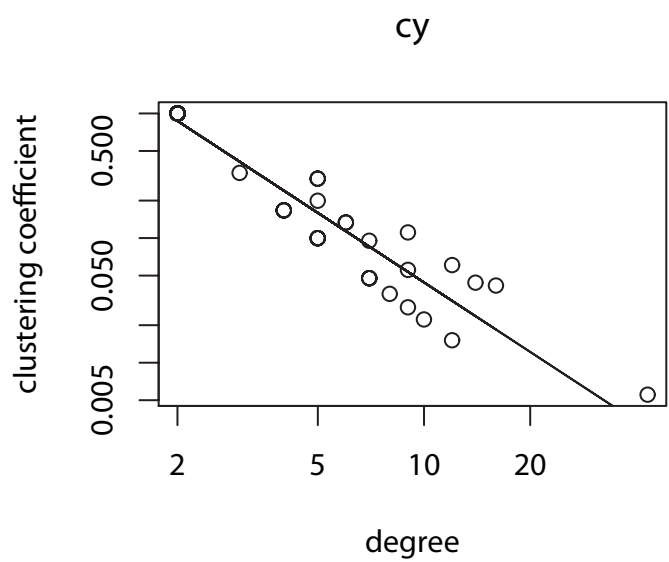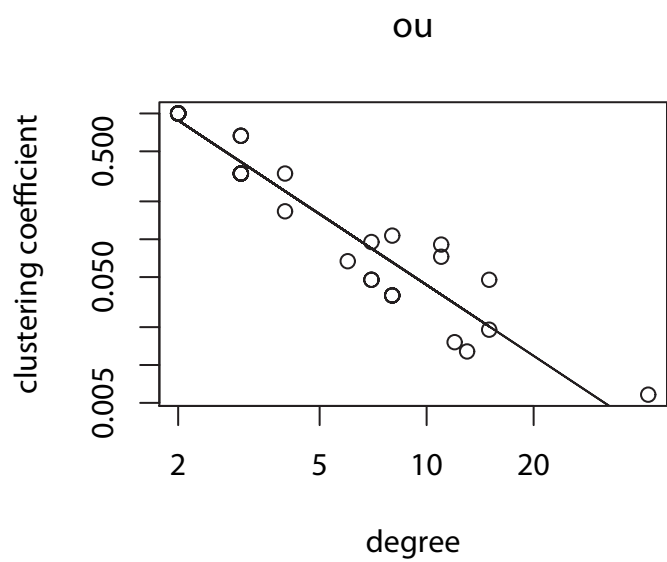

mw

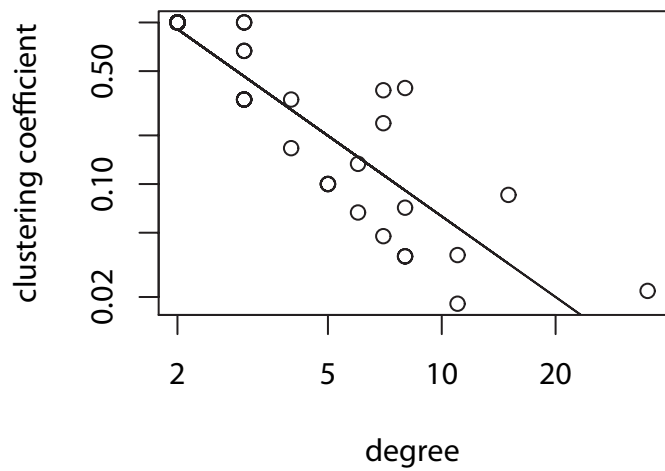

pt

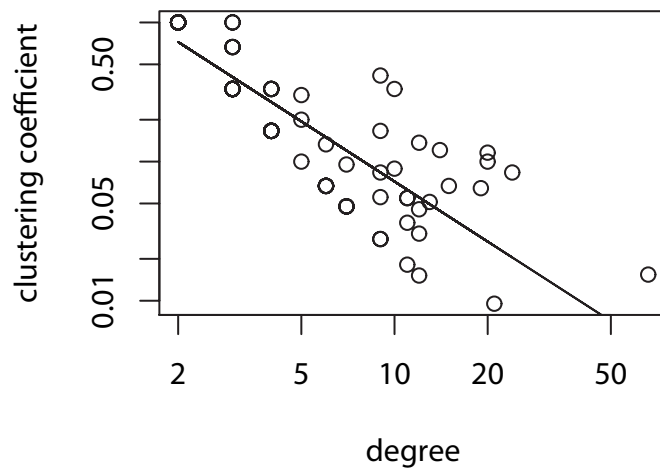

at

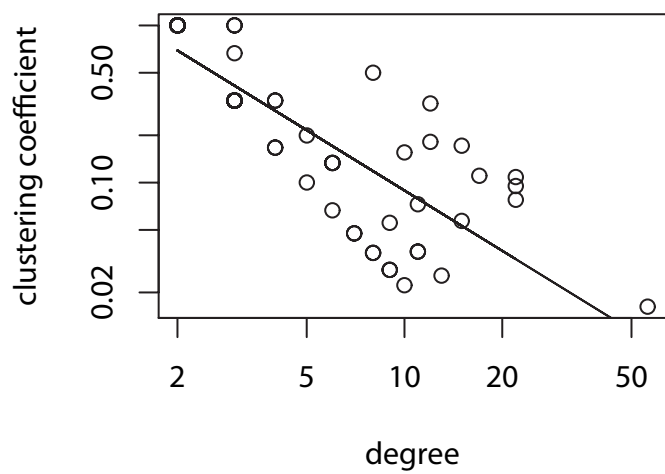

os

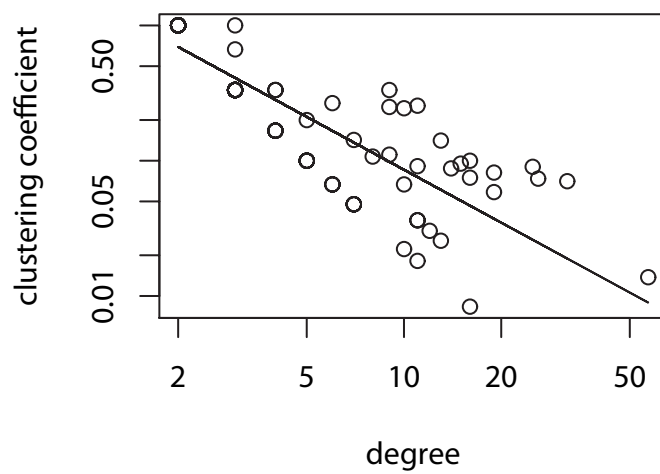

dm

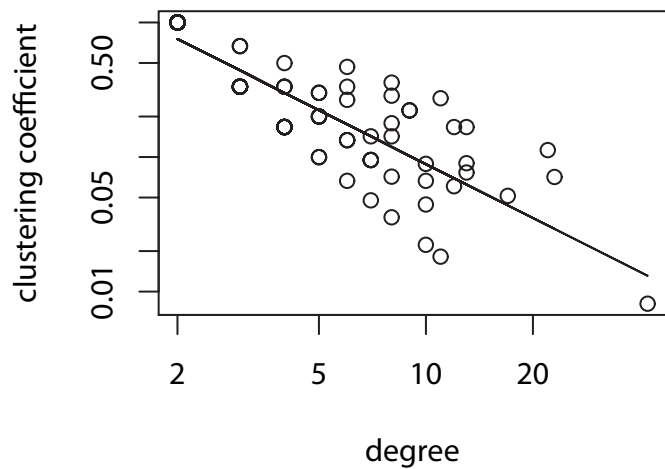

dd

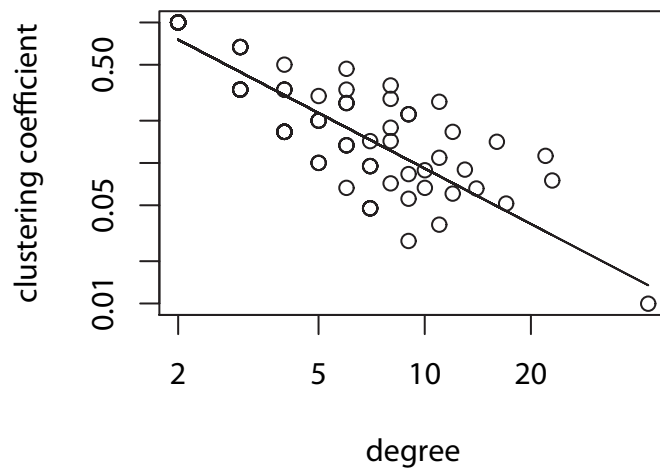

do

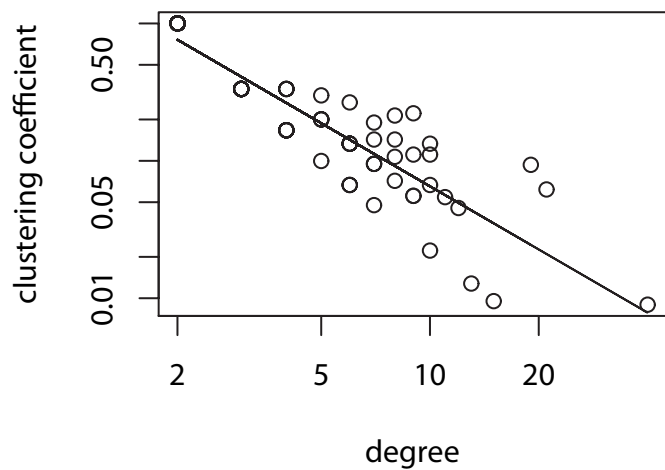

ag

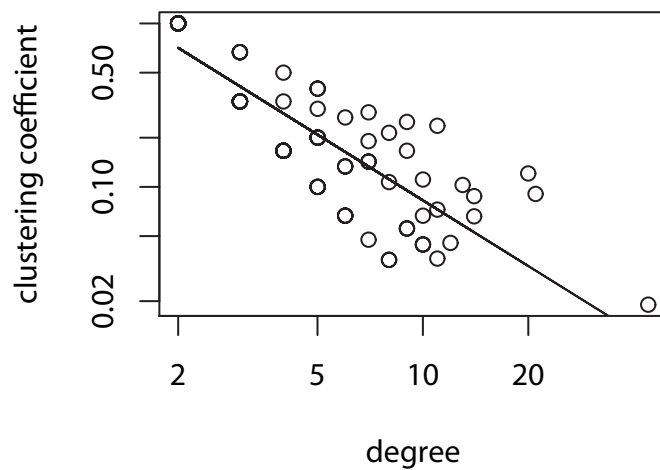

ax

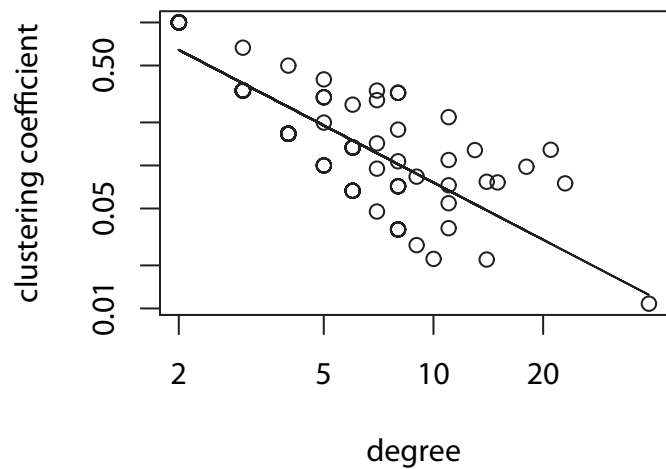

ai

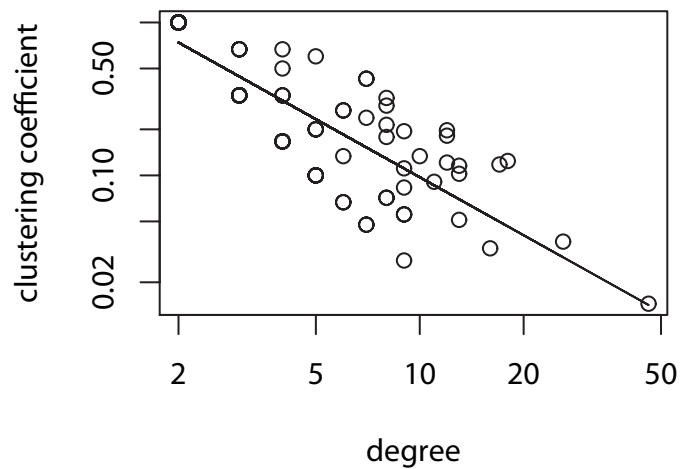

om

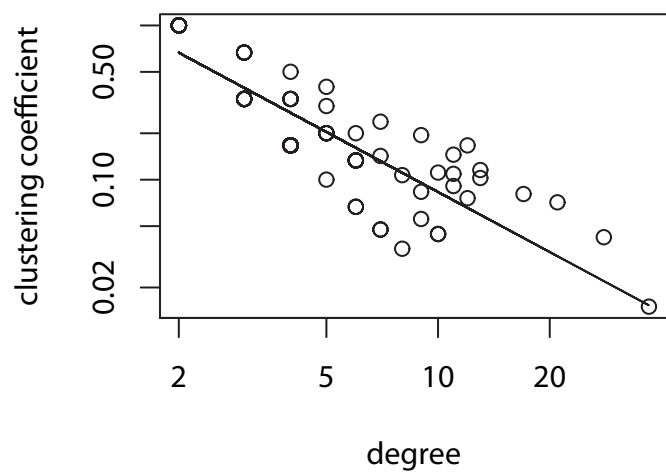

ol

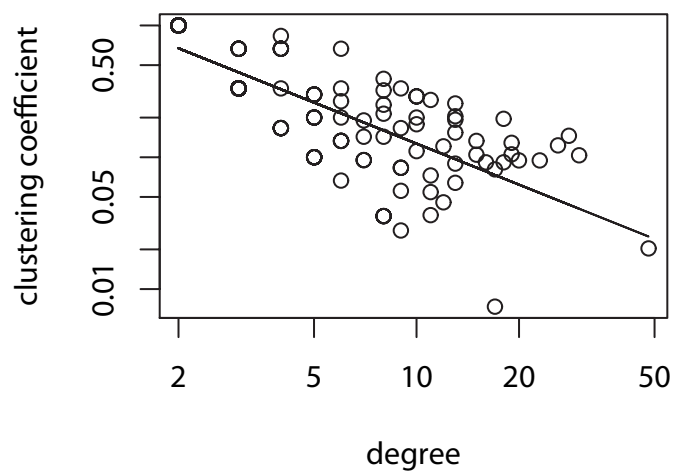

gc

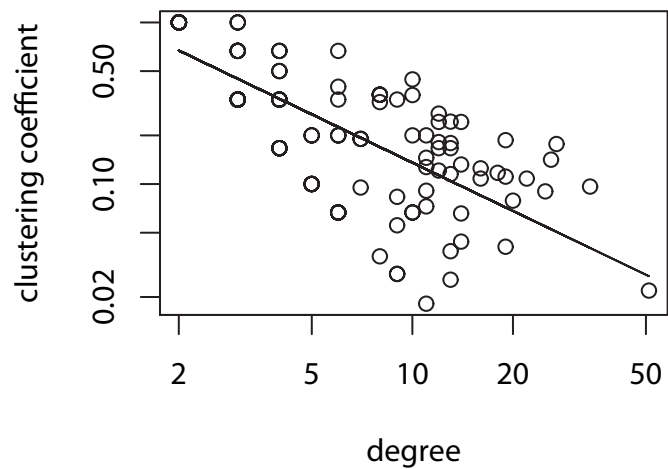

to

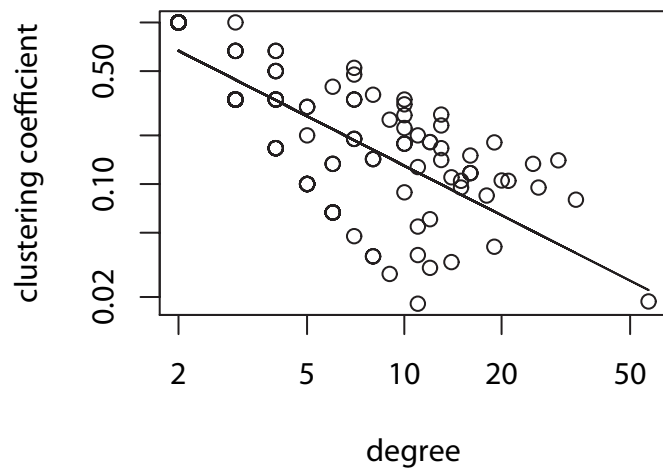

tn

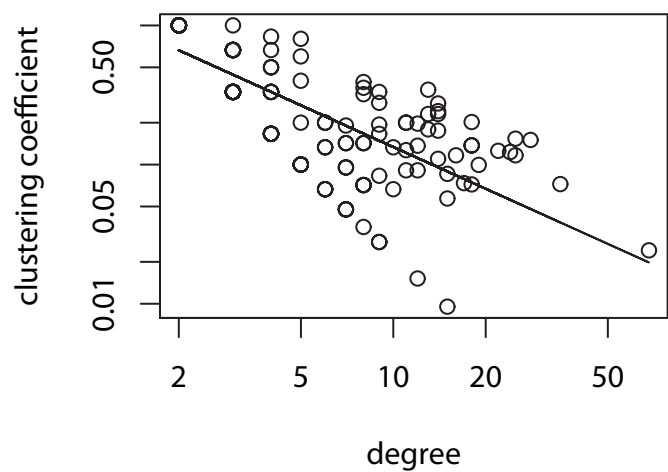

da

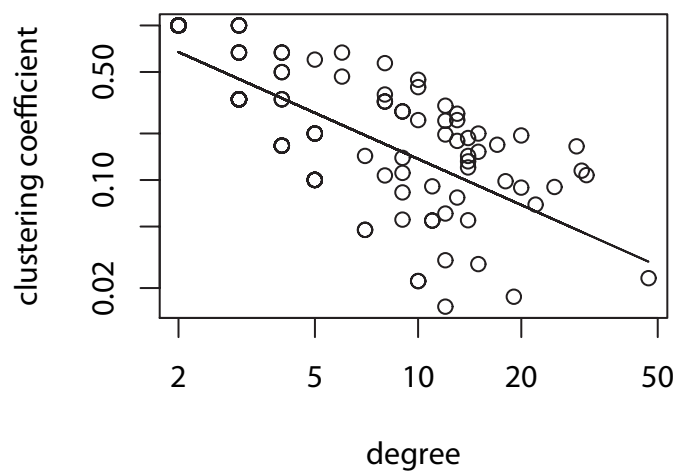

xn

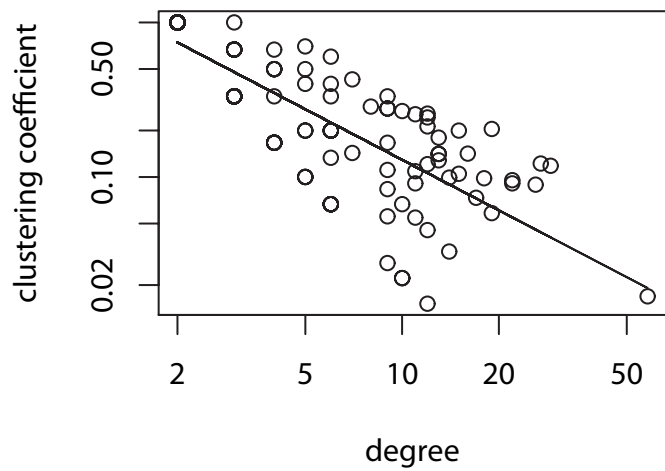

gg

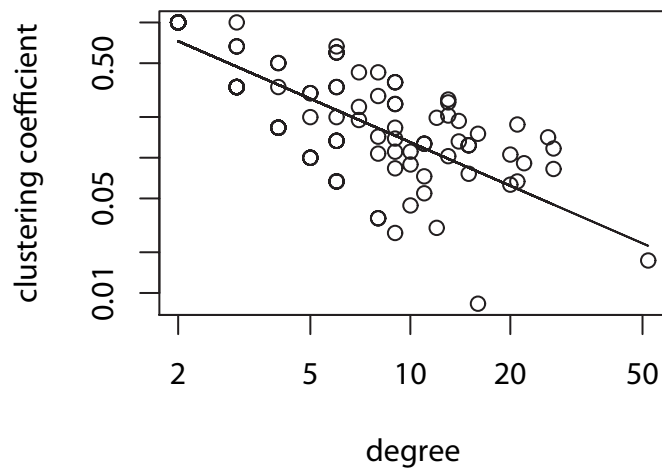

mm

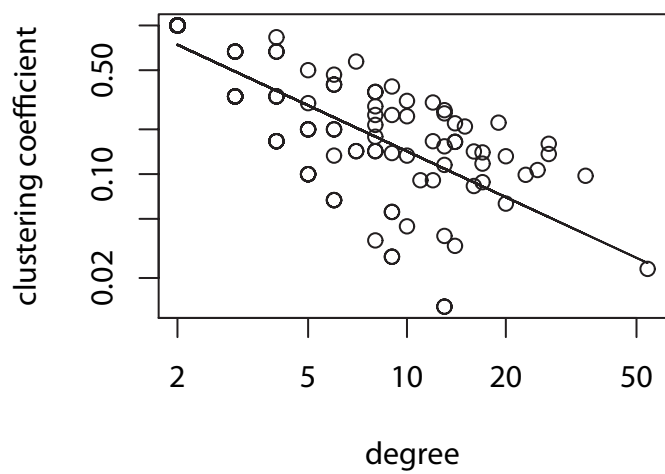

ru

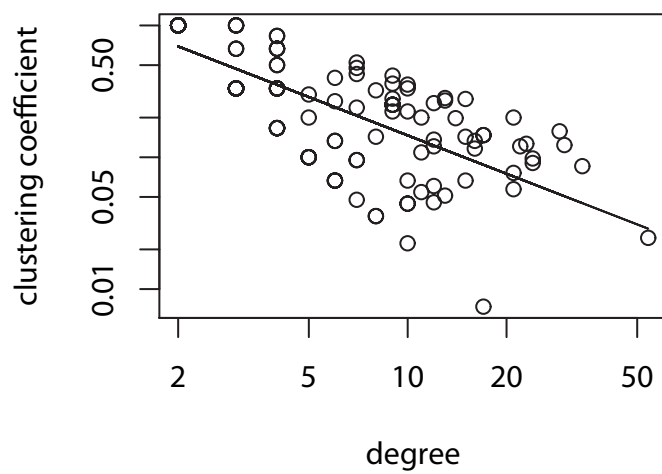

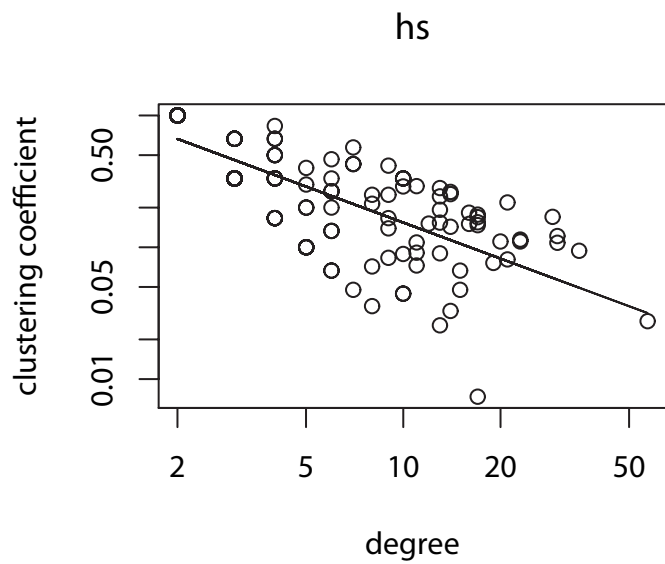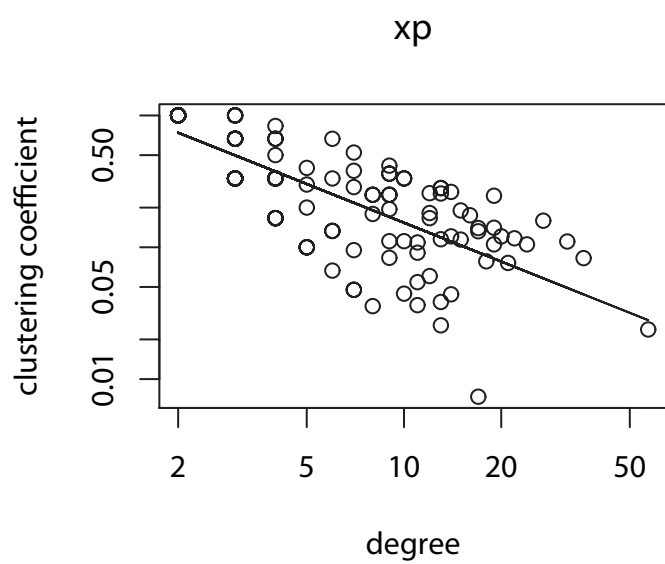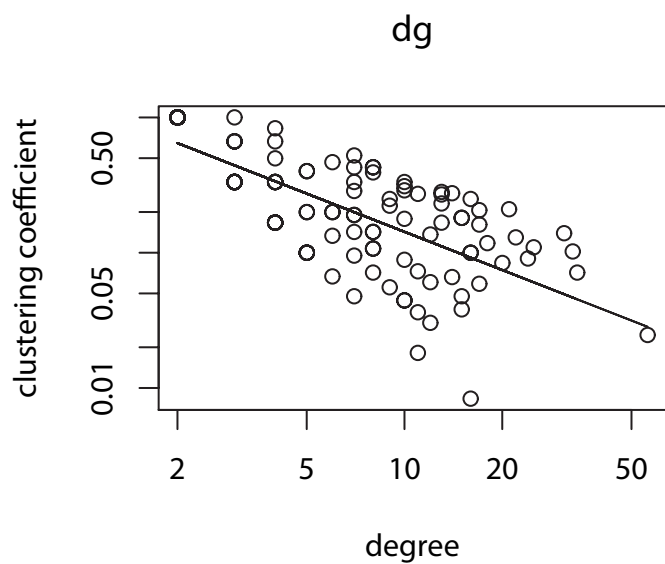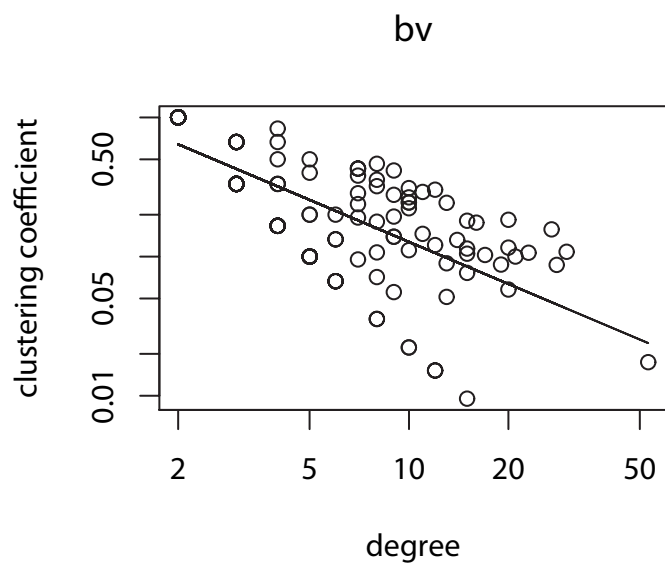

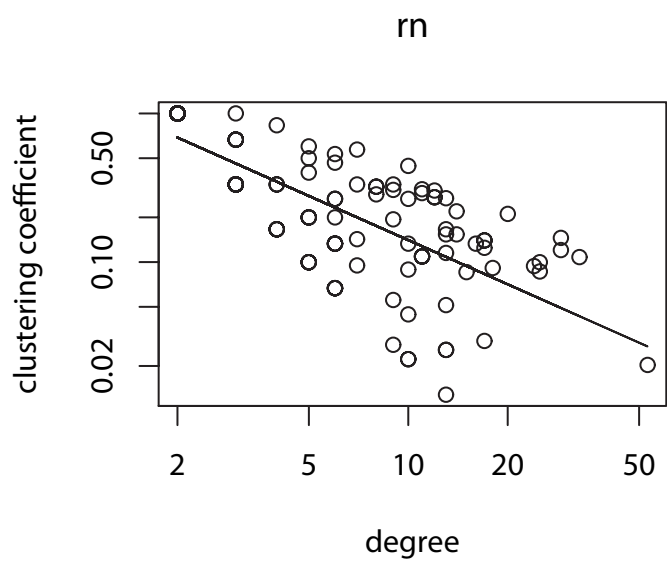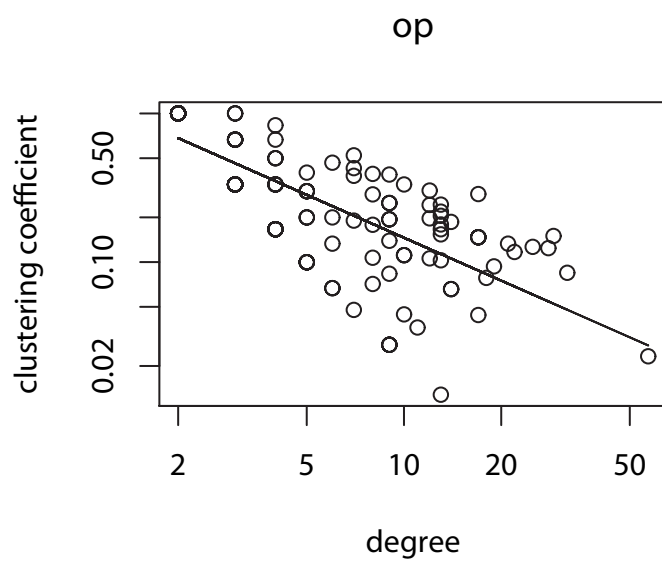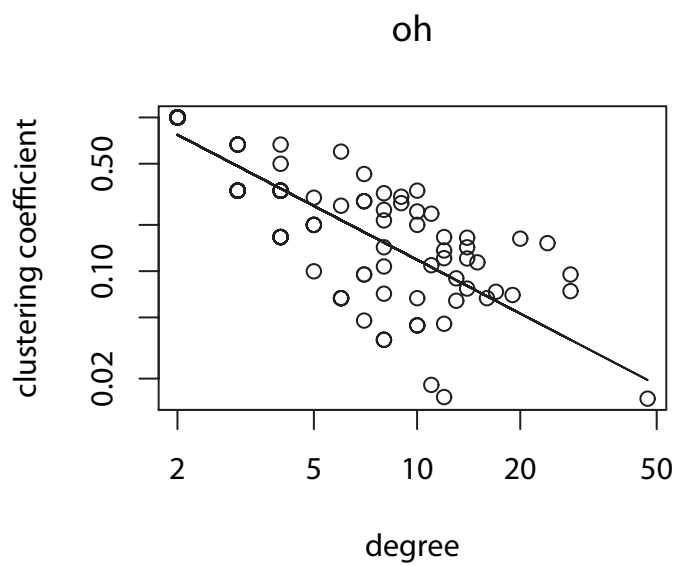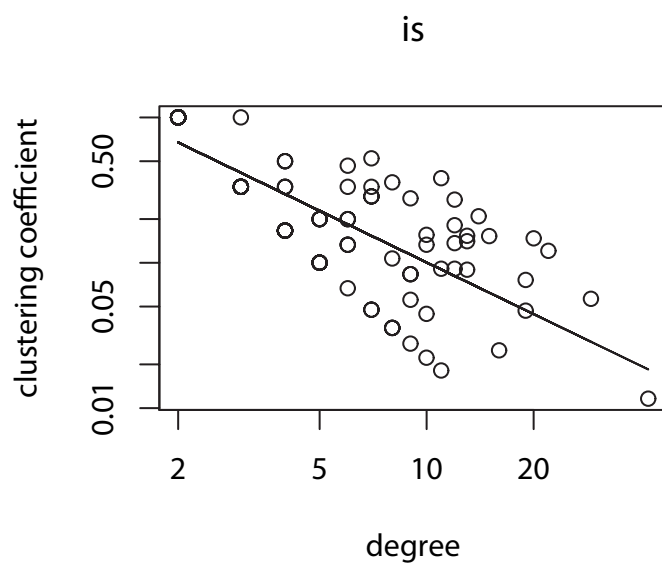

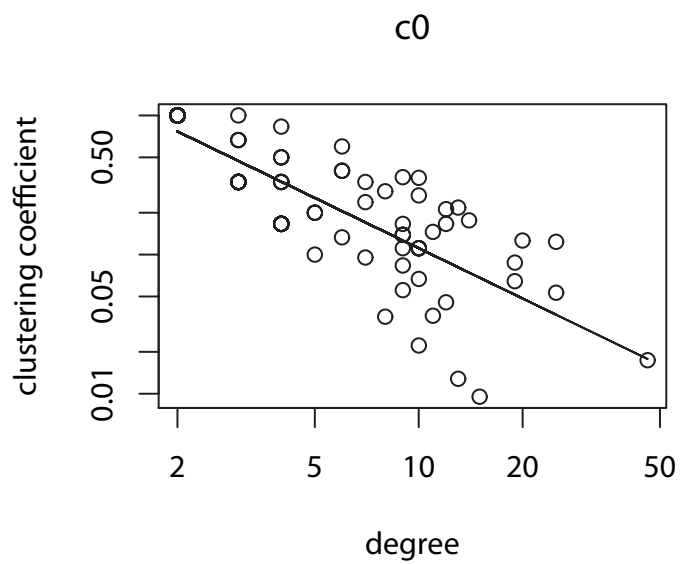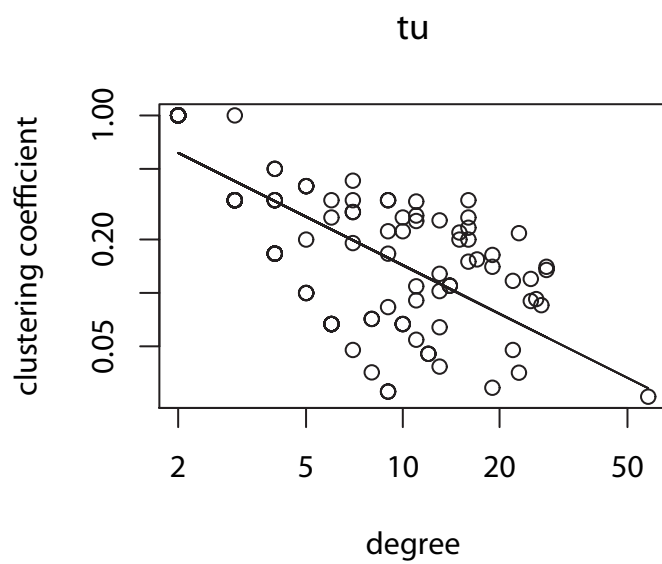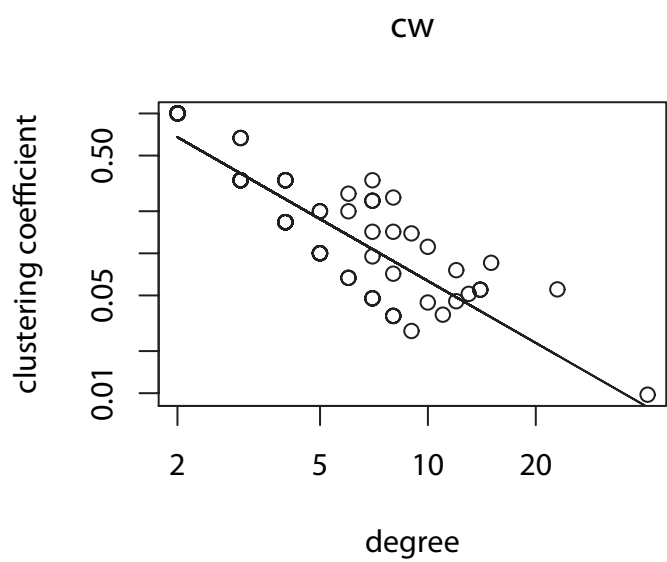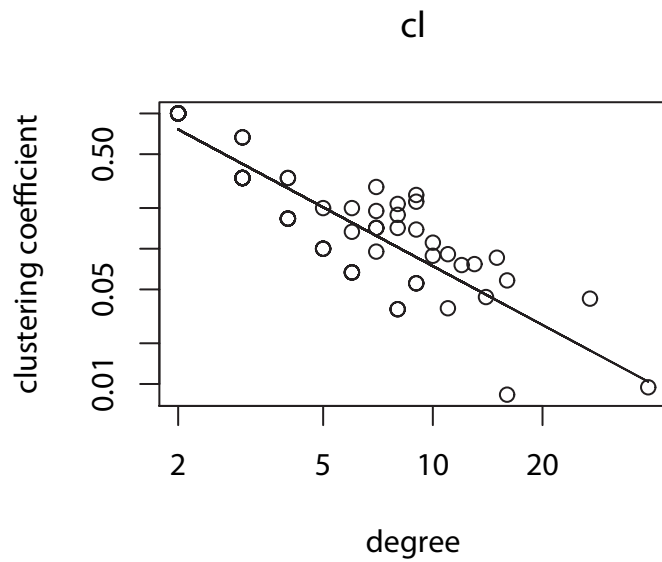

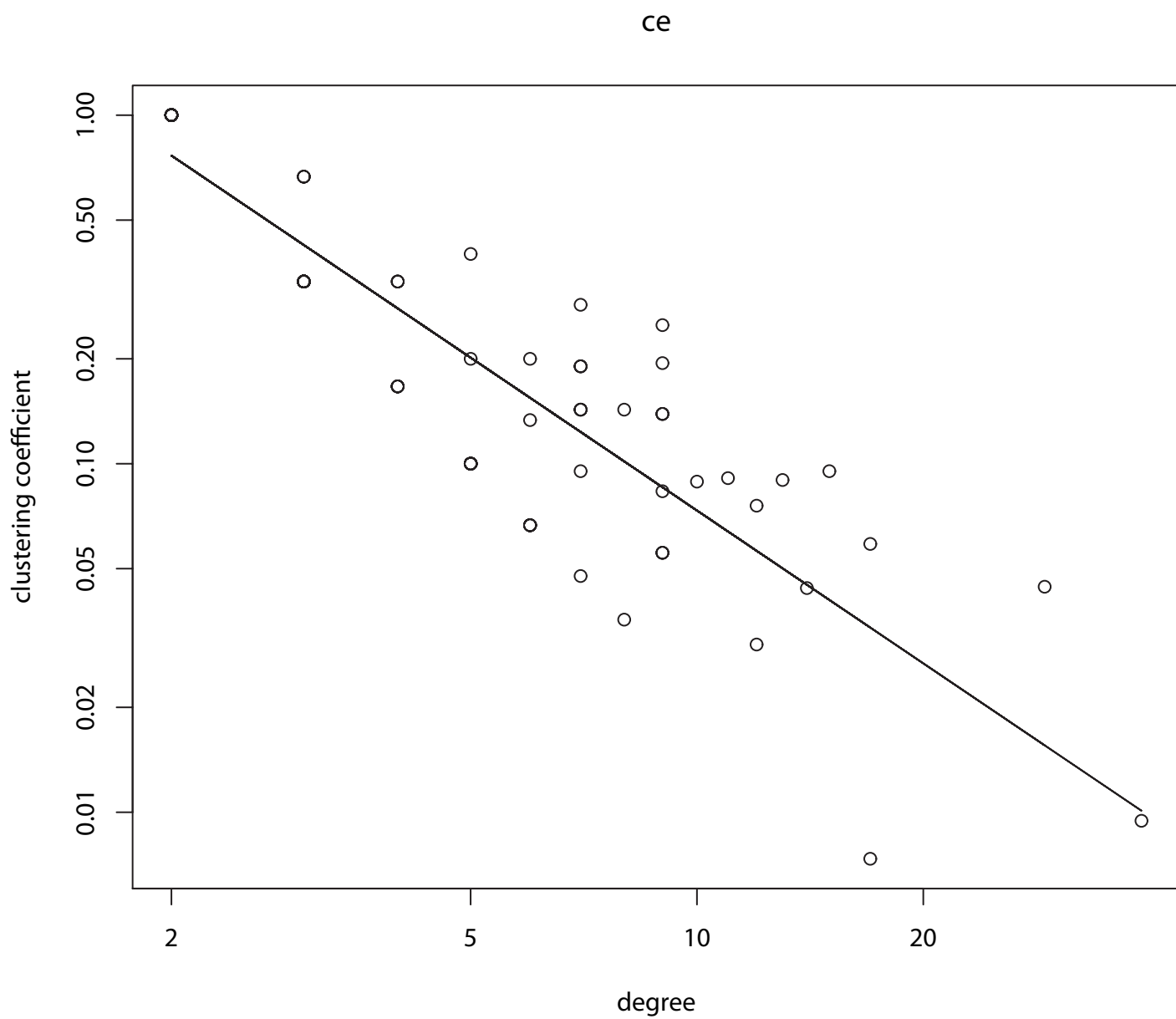

Supplement: Additional File 3 — The dependence of clustering coefficient on node degree in the 77 domain bigram networks. The log-log plots of clustering coefficient versus the degree of node in networks. [file 1471-2148-11-242-S3.PDF]
